# Potential modulations in flatland: near-infrared sensitization of MoS_2_ phototransistors by a solvatochromic dye directly tethered to sulfur vacancies

**DOI:** 10.1038/s41598-019-53186-2

**Published:** 2019-11-13

**Authors:** Simon Dalgleish, Louisa Reissig, Yoshiaki Shuku, Giovanni Ligorio, Kunio Awaga, Emil J. W. List-Kratochvil

**Affiliations:** 1 Department of Chemistry and IRCCS, Nagoya University, Furo-cho, Chikusa, 464-8602 Nagoya Japan; 2 Institut für Physik, Institut für Chemie & IRIS Adlershof, Humboldt-Universität zu Berlin, Brook-Taylor-Str. 6, 12489 Berlin, Germany; 3 Institute of Experimental Physics, Freie Universität Berlin, Arnimallee 14, 14195 Berlin, Germany; 4 Helmholtz-Zentrum Berlin für Materialien und Energie GmbH, Brook-Taylor-Str. 6, 12489 Berlin, Germany

**Keywords:** Nanophotonics and plasmonics, Nanosensors

## Abstract

Near-infrared sensitization of monolayer MoS_2_ is here achieved *via* the covalent attachment of a novel heteroleptic nickel *bis*-dithiolene complex into sulfur vacancies in the MoS_2_ structure. Photocurrent action spectroscopy of the sensitized films reveals a discreet contribution from the sensitizer dye centred around 1300 nm (0.95 eV), well below the bandgap of MoS_2_ (2.1 eV), corresponding to the excitation of the monoanionic dithiolene complex. A mechanism of conductivity enhancement is proposed based on a photo-induced flattening of the corrugated energy landscape present at sulfur vacancy defect sites within the MoS_2_ due to a dipole change within the dye molecule upon photoexcitation. This method of sensitization might be readily extended to other functional molecules that can impart a change to the dielectric environment at the MoS_2_ surface under stimulation, thereby extending the breadth of detector applications for MoS_2_ and other transition metal dichalcogenides.

## Introduction

Within the class of 2D materials, transition metal dichalcogenides (TMDCs), such as MoS_2_, have been shown to be excellent candidates for next generation electronic and optoelectronic applications since they show strong absorption for visible light (*α* > 10^6^ cm^−1^)^1^ and high charge carrier mobilities (*μ* > 100 s cm^2^ V^−1^ s^−1^) have been recorded^2,3^, even for single monolayer flakes. In contrast to graphene, MoS_2_ possesses a band-gap, direct in the case of monolayers, and has shown high on-off ratios (>10^8^) in field-effect transistor (FET) devices^4,5^ thereby holding much promise for switching applications. Under optical stimulation, MoS_2_-based FETs have shown very high responsivities (*Ɍ* = 880 AW^−1^)^6^ across the visible region and various studies have sought to extend the sensitivity into the near infrared (NIR) region, and beyond, through charge transfer mechanisms between MoS_2_ and *i*.*a*. other 2D materials^7^, organic dyes^8^ or quantum dots – both in direct contact with the MoS_2_^9^ or *via* a thin interlayer of TiO_2_^10^. Especially in the latter case, extremely high responsivities of the order 10^6^ AW^−1^ have been reported, owing to the passivation of the MoS_2_ surface by the TiO_2_ interlayer which effectively suppressed the direct interaction between QDs and the MoS_2_ channel, thereby preserving the intrinsic high on-off ratio of the MoS_2_, while allowing photocurrent gain *via* charge transfer across the interlayer.

In TMDC-based FET devices, since the active layer can be reduced to a single monolayer, the channel conductance is sensitive not just to the nature of the gate dielectric surface, for a given field, but also to the medium to which the opposed surface is exposed, *i*.*e*. adsorbed atoms, molecules or ions affect directly the channel between the source and drain electrodes without screening from a bulk semiconductor overlayer^11^. As such, monolayer TMDC-FETs have been shown to be extremely sensitive to the ambient conditions under which they are measured^12^, which can severely affect the electrical properties of devices but, if controlled^10^, also makes them an excellent platform for sensing applications.

Recently, a method to tune the conductance in planar 2- and 3-terminal devices of MoS_2_ (a representative TMDC) has been demonstrated, based on the covalent binding of thiol-based molecules at sulfur vacancies that exist (or can be introduced) as defects in the MoS_2_ structure^13^. By varying the end group of the thiol between electron rich and electron poor groups, it is possible to modify the carrier density in the MoS_2_, and thereby the threshold voltage (*V*_T_) of charge accumulation in the channel, in a controlled and permanent manner. To date, such studies have been limited to passive thiol molecules that are insensitive to external stimuli, thus the response of the devices is limited to the properties of the TMDC, such as its range of light absorption (*λ*_cutoff_ ≈ 700 nm for MoS_2_) and non-specific interaction with adsorbates. The use of functional molecules as covalently-bound addressable surface sensitizers for TMDC-based planar devices would allow for modulation of the channel conductance under external stimuli *via* a local and oriented change to the dielectric environment close to the TMDC channel, thereby providing a versatile platform for realizing a range of sensor devices that combine the superior (opto)electrical properties of TMDCs with the sensitivity and selectivity of organic/bio functional molecules. Such a concept has previously been demonstrated for application in biosensing to detect specific binding events of charged antigens to antibodies physadsorbed to the MoS_2_ channel^14,15^. However, to date, no sensitization *via* a unimolecular dipole change has yet been demonstrated.

In this study we demonstrate the principle of near-infrared (NIR) sensitization of monolayer MoS_2_ phototransistors *via* direct covalent attachment of a heteroleptic nickel *bis*-dithiolene (NiDT) chromophore to the MoS_2_ surface. NiDTs are attractive targets as sensitizer dyes as they characteristically show high thermal and photochemical stability, as well as strong optical absorption in the NIR (*ε* = 10^4^–10^6^ M^−1^ cm^−1^, *λ*_max_ = 700–1500 nm) that is highly tuneable through rational ligand design^16^. By designing a NiDT dye with a single pendant disulfide unit, capable of binding to sulfur vacancies in the MoS_2_, dye-sensitization is shown to yield MoS_2_ phototransistors with an extended range of photodetection up to 1500 nm (0.83 eV), due to the oriented charge rearrangement within the dye molecule upon photoexcitation.

## Results and Discussion

**Figure 1:**
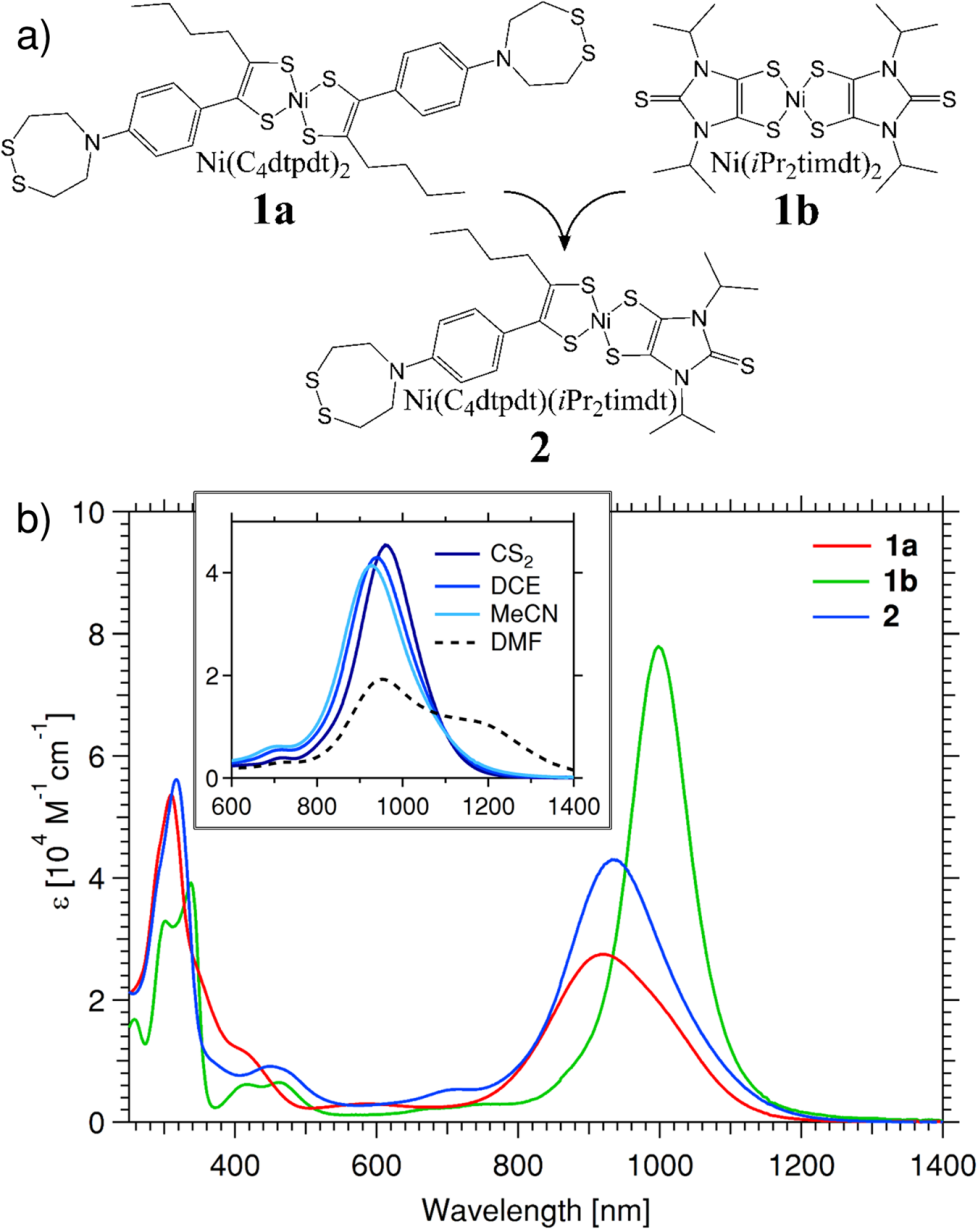
(**a**) Molecular structure of heteroleptic Ni(C_4_dtpdt)(*i*Pr_2_timdt) (**2**), synthesized by a ligand scrambling reaction between its two homoleptic precursors Ni(C_4_dtpdt)_2_ (**1a**) and Ni(*i*Pr_2_timdt)_2_ (**1b**); (b) UV/Vis/NIR absorption spectra of **2**, compared to (**1a** and **1b)** recorded in dichloromethane, showing intermediate values of both peak absorption (*λ*_max_) and extinction coefficient (*ε*); (inset: red shift in *λ*_max_ for **2** with decreasing solvent polarity, indicating negative solvatochromism).

In a first step, a dithiolene ligand was designed that could serve as an anchor to chalcogen vacancies in TMDCs. The target ligand, 1-phenyl-[1,2,5]-dithiazepane-hex-1-ene-1,2-dithiolate (C_4_dtpdt), contained a pendant dithiazapane group that could potentially allow a bi-podal binding to the TMDC surface *via* ring opening at the disulfide bond^17^, thereby enhancing the binding stability. The synthetic route to the ligand precursor is described in detail in the Supporting Information online, and was achieved in 5 steps with an overall yield of >30%. In order to achieve an oriented change to the potential landscape at the MoS_2_ surface from the sensitizer molecule under optical stimulation, the target dye was heteroleptic, thus capable of binding only at one terminus. Heteroleptic NiDTs can generally be achieved through a ligand scrambling reaction between two dissimilar homoleptic complexes in an appropriate oxidation state^18^. Scrambling with neutral Ni(*i*Pr_2_timdt)_2_ (**1b**) (a complex with an exceptionally high molar extinction coefficient)^19^ was successful and the heteroleptic Ni(C_4_dtpdt)(*i*Pr_2_timdt) (**2**) could be isolated in *ca*. 20% yield from its homoleptic precursors, as shown in Fig. 1a.

The electronic characteristics of **2**, as studied by absorption spectroscopy and cyclic voltammetry, were shown to be intermediate between **1a** and **1b** (Table 1, Fig. S1, ESI), with a peak absorption for **2** at *λ* = 935 nm in dichloromethane (DCM), as shown in Fig. 1b. The complex showed weak negative solvatochromism (Δ*λ* = 35 nm (392 cm^−1^) between CS_2_ and MeCN) that scaled well with the refractive index of the solvent (Table S1, Fig. S2, ESI), suggesting some “push-pull” character to the electronic structure of the molecule^18^. Absorption spectroscopy in certain high donor number (DN) solvents (DMF, DMSO) showed the presence of a new peak at *ca*. 1200 nm that increased in intensity with time, at the expense of the main NIR peak. The location and progression of this peak was similar to that of the reduced species, studied under spectroelectrochemistry (*c*.*f*. Fig. S3, ESI), suggesting a spontaneous reduction of the complex in high DN solvents^20^. From X-ray structural analysis of **2** (Tables S2 and S3, Fig. S4, ESI), the bond lengths of the core NiSCCS rings are consistent with a greater dithiolate (“pull”) character for the C_4_dtpdt ligand and a greater dithione (“push”) character for the *i*Pr_2_timdt ligand. This assignment is further supported by the slight reduction/extension of the C-C and S-Ni bond lengths in the respective ligands of **2**, compared those of the symmetric parents **1a**/**1b** where the electron density is equally shared over the two ligands. Such assignment leads to the conclusion that the C_4_dtpdt ligand makes a greater contribution to the HOMO of **2**, with the LUMO residing more on the *i*Pr_2_timdt ligand, giving the optical transition some degree of intramolecular charge transfer (ICT) character. However, the intermediate electrochemical and optical properties of **2**, compared to its parents **1a** and **1b** suggest the dominant resonance form is a π-delocalized core with the optical transition principally *π*-π* in origin^18^.

**Table 1:** Electronic characteristics of **2**, compared to those of **1a** and **1b**, as determined by absorption spectroscopy and cyclic voltammetry (^§^ denotes irreversible process – *E*_1/2_ based on relative onset, as described in the ESI) quoted against the Fc/Fc^+^ redox couple; HOMO & LUMO levels, as well as the transport gap (Δ*E*_g_) estimated by the empirical method of Sworakowski *et al*.^34^.

	*λ*_max_ (ε × 10^4^)	${E}_{1/2}^{{\rm{red}}2}$	${E}_{1/2}^{{\rm{red}}}$ (LUMO)	${E}_{1/2}^{{\rm{ox}}}$(HOMO)	Δ*E*_g_
	nm (M^−1^cm^−1^)	V	V (eV)	V (eV)	eV
**1a**	919 (2.75)	−1.48^§^	−0.67 (−4.03)	0.35 (−5.19)	1.16
**1b**	998 (7.80)	−1.08	−0.62 (−4.09)	0.30^§^ (−5.13)	1.04
**2**	935 (4.30)	−1.27	−0.65 (−4.06)	0.33 (−5.17)	1.11
**MoS**_**2**_	—	—	(−4.15)	(−6.26)	2.11

Representative values for MoS_2_ are given for comparison^30^.

In order to demonstrate dye sensitization of MoS_2_ with **2**, commercial large-area CVD-grown monolayer MoS_2_ films, grown on sapphire, were used (Fig. S5, ESI). Dye-sensitization was achieved in a similar manner to that reported by Sim *et al*.^13^, whereby thermal annealing was used to introduce sulfur vacancies in the MoS_2_, which was then soaked in a solution of **2**, followed by copious washing with fresh DCM. In all cases, the sensitized films (MoS_2_-**2**) were compared to as-received samples (MoS_2_), as well as control samples MoS_2_-C and MoS_2_-**1b**, processed in an identical manner to MoS_2_-**2**, except that they were soaked in pure DCM or **1b** in DCM, respectively.

**Figure 2:**
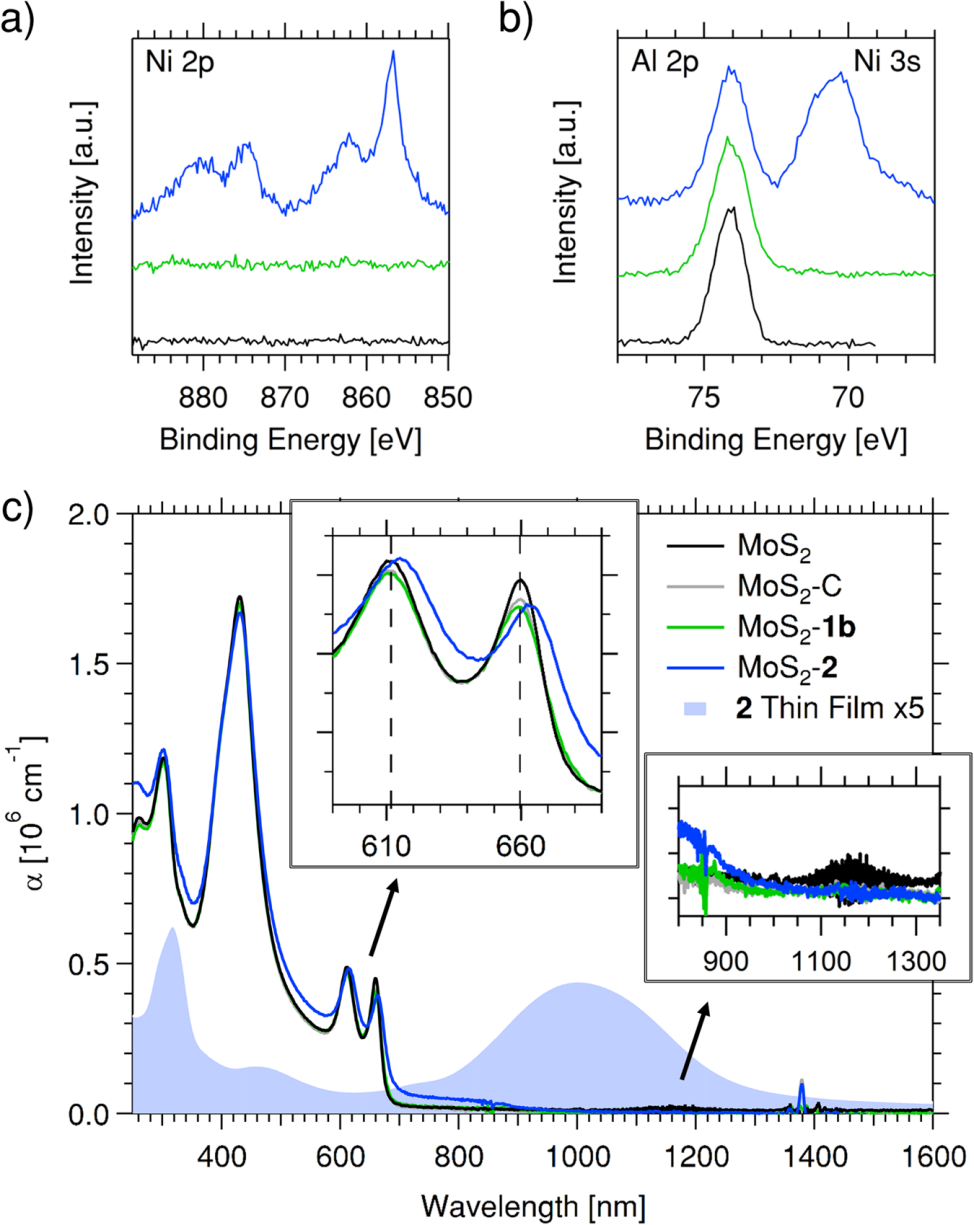
Spectroscopic characterization of various MoS_2_ films: (**a**,**b**) XPS spectra of Ni 2p region (**a**) and Ni 3 s region, including Al 2p peak from substrate (**b**) for MoS_2_-**2**, compared to MoS_2_-C and MoS_2_-**1b** (data offset for clarity, colour scheme as for (**c**)); (**c**) thin film absorption spectra (absorption coefficient *α* based on an estimated monolayer thickness of *d* = 0.85 nm), compared to the thin film spectra of **2** (*d* = 50 nm), showing no detectable NiDT peak for MoS_2_-**2** (right inset), but a red shift in the MoS_2_ excitonic transitions (left inset) (note: the slight y-offset and noise at 1150 nm in the MoS_2_ spectrum was common to as received samples – see ESI for further discussion).

Figure 2 shows the spectroscopic characterization of the various MoS_2_ films. The binding of **2** to MoS_2_ is confirmed by X-ray photoelectron spectroscopy (XPS) of MoS_2_-**2**, which solely showed the distinct Ni elemental signature by the rising of Ni 2p peaks (Fig. 2a) as well as the Ni 3s peak in the Al 2p region of the sapphire substrate (Fig. 2b). Ni was not observed in either control sample MoS_2_-C or MoS_2_-**1b**. Since the chemical composition and energy levels of **1b** and **2** are very similar, it can be concluded that the binding of **2** to MoS_2_ is achieved *via* the disulfide moiety of the dithiazepane, rather than by other mechanisms of association, such as serendipitous adsorption, or charge transfer (CT) complex formation. This is confirmed by the increase of the S signal, relative to Mo, in MoS_2_-**2** compared to MoS_2_-C and MoS_2_-**1b** in which the signal ratio stays almost constant (*c*.*f*. Fig. S6 and Table S4, ESI).

Under UV/Vis/NIR spectroscopy (Fig. 2c), no characteristic NIR absorption could be detected for MoS_2_-**2**, and the spectra of MoS_2_-**2**, MoS_2_-C and MoS_2_-**1b** were almost identical in the region beyond 1000 nm, as shown in the NIR inset of Fig. 2c,suggesting a low surface coverage of **2** upon binding. Nevertheless, a distinct and reproducible change to the MoS_2_ absorption could be seen in the position of the A and B excitonic transitions, which were shifted to lower energy, with a broadening of the onset of light absorption, as shown in the visible region inset of Fig. 2c. This was not due to thermal annealing or DCM soaking, as shown by the near identical peak positions for the control samples compared to the as-received sample and thus might represent some electronic change to the MoS_2_ following sensitization (*vide infra*).

The functionality of the dye-sensitized MoS_2_ was assessed in FET devices, studied under photocurrent action spectroscopy. MoS_2_ was transferred to bottom-gate/bottom-contact (BGBC) Si/SiO_2_ transistor substrates by surface-energy-assisted transfer using polystyrene as a support, as reported by Gurarslan *et al*.^21^ (*c*.*f*. Fig. S7, ESI). In an attempt to reduce the strain in the MoS_2_, induced by a bottom contact geometry, ultra-thin Au source/drain electrodes (*d* = 9 nm) were initially used, fabricated on a mixed amine/thiol self-assembled monolayer adhesion layer^22^. The integrity of such electrodes was confirmed by atomic force microscopy and 4-point probe conductivity measurements, yielding a RMS roughness *R*_*q*_ < 1 nm and a sheet resistance *R*_*s*_ < 10 Ω/sq. For unannealed devices using this architecture, transistor analysis of the directly transferred films yielded a relatively low carrier mobility of the order *μ*_*e*_ ≈ 10^−3^ cm^2^ V^−1^s^−1^. The mobility values were generally improved following thermal annealing at 250 °C and subsequent soaking in DCM or **2** in DCM (MoS_2_-C and MoS_2_-**2**) to *μ*_*e*_ ≈ 10^0^ cm^2^ V^−1^s^−1^, in both cases showing a threshold voltage close to −10 V. Due to the relatively broad spread in data, and low sample numbers tested, no statistical relevant difference between the electrical properties of MoS_2_-C and MoS_2_-**2** was observed.

**Figure 3:**
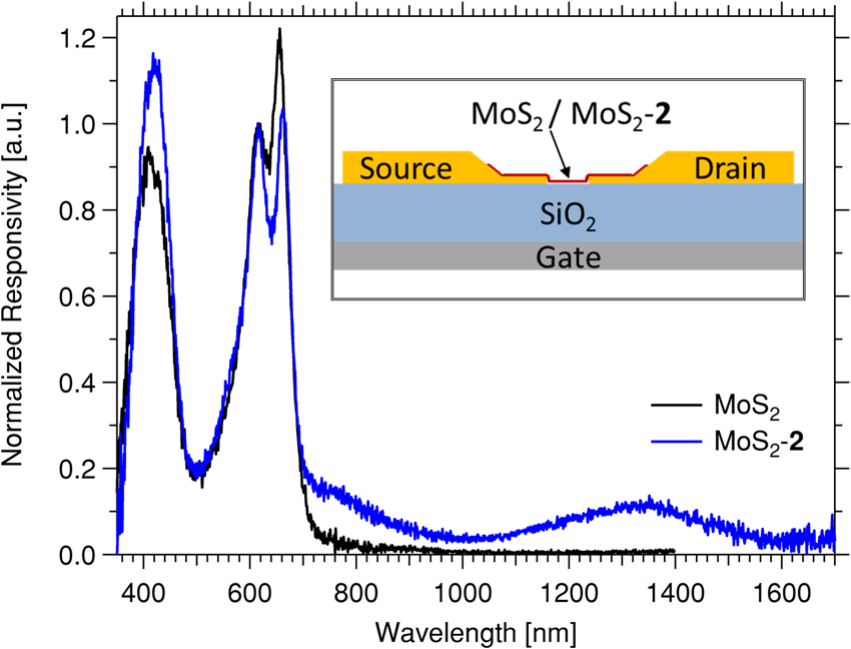
Wavelength-dependent photoconductivity of MoS_2_-**2**, compared to unsensitized MoS_2_-C (normalized to the B excitonic process), showing clear photocurrent generation in the NIR for MoS_2_-**2**. BGBC transistor substrates were used with ultra-thin Au electrodes as source and drain (*L* = 30 μm, *W* = 1 mm), as depicted in the inset, and the action spectra were recorded by lock-in methods at 263 Hz with *V*_DS_ = 10 V, *V*_GS_ = 0 V.

Figure 3 shows the wavelength-dependent photoconductivity (action spectra) of MoS_2_ phototransistors with and without sensitization by **2** over a wavelength range 350 nm – 1700 nm. The three excitonic processes of MoS_2_ are represented in the visible region, with their onset of photoconductivity limited to *ca*. 700 nm (1.77 eV), consistent with previous reports^23^. In the case of MoS_2_-**2**, the relative heights of these processes are changed, with a marked decrease in the relative contribution of the A excitonic process. Most notably, a new band, centred around 1300 nm, is apparent for MoS_2_-**2**, that is not observed in MoS_2_. This NIR photoresponse is observed in both scanning directions, and its magnitude remains stable over 1 hr of continuous measurement at 1300 nm. While the wavelength of this process does not correspond to that of **2**, either in solution (*ca*. 940 nm, *c*.*f*. Fig. 1b), or as a thin film (*ca*. 1000 nm, *c*.*f*. Fig. 2c), it is similar to the peak absorption of the reduced complex [**2**]^−^ studied under spectroelectrochemistry (*c*.*f*. Fig. S3, ESI). Further investigations are necessary to determine whether this redox change is effected in the device under operation, or is symptomatic of the binding mechanism and/or the redox sensitivity of **2** under certain environments (*c*.*f*. absorption spectra in DMSO and DMF) – certainly the presence of a charged molecule on the surface of the MoS_2_ might account for the shift in the A and B absorption peaks observed for MoS_2_-**2** (*c*.*f*. Fig. 2c).

In this architecture, measurements under steady-state illumination were complicated by an extremely long-lived persistent photocurrent (PCC) contribution, that did not fully recover for > 10 hrs following visible or NIR illumination. Such an effect is commonly observed for MoS_2_ devices fabricated on SiO_2_ substrates^6^, the origin of which has been traced to carrier trapping both at the MoS_2_/SiO_2_ interface^24,25^, as well as within the MoS_2_^25,26^. By shortening the channel length (*L* = 5 μm), using pre-fabricated interdigitated electrode array (IDA) BGBC devices, the recovery time was greatly reduced. It should be noted that the use of this architecture also caused a reduction in carrier mobility to *μ*_*e*_ ≈ 10^−2^–10^−3^ cm^2^ V^−1^s^−1^, even for the annealed films, which was likely due to the increased strain and possible damage caused by the relatively thick bottom contact electrodes over which the MoS_2_ was spread (Fig. S8, ESI). For such devices, the photocurrent response under illumination at 639 nm and 1310 nm (stimulating MoS_2_ and **2**, respectively) both show a sub-linear increase with increasing light intensity, thus representing a drop in absolute responsivity towards higher light powers, though the relative responsivities at the two wavelengths remain approximately constant, as shown in Fig. S9, ESI. This suggests a constant contribution of **2** to the photocurrent response, rather than a transient effect of charging/discharging under illumination.

**Figure 4:**
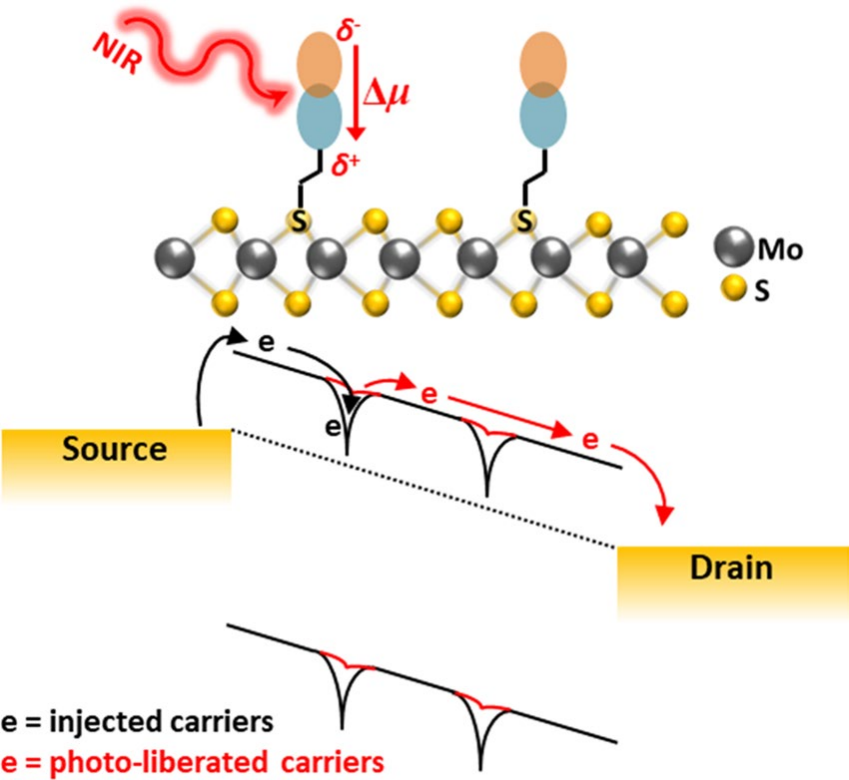
Proposed mechanism of NIR photocurrent generation. Processes occurring in the dark are shown in black, while processes due to NIR stimulation are shown in red. Photoexcitation of the solvatochromic sensitizer dye leads to a dipole change at the surface of the MoS_2_, thereby liberating trapped charges and preventing their (re-)trapping.

Considering the origin of photoresponse in the NIR, we propose a mechanism based on a dipolar change at the MoS_2_ surface that acts to liberate trapped charges within the MoS_2_ and/or limit their re-trapping. Coulomb interactions between TMDCs with polar molecules on their surface (as well as surface states on the substrate)^27^ have been shown to cause a local effective self-energy correction in the TMDC and thereby cause local variations of the bandgap^28^. Since the dye molecules are directly tethered to sulfur vacancies, which are a known locus of electron trapping and which can further scatter mobile carriers^11,29^, any change to the dipole moment of the dye affects directly the trap depth and trapping lifetime due to a change in the local dielectric environment of the MoS_2_. Photoexcitation of **2** is expected to cause a redistribution of electron density away from the anchoring C_4_dtpdt ligand (*vide supra*), thus yielding a *δ*^+^ close to the MoS_2_ surface. This local electrostatic change from **2** under NIR illumination effectively reduces the trap depth at the sulfur vacancies, liberating trapped charge carriers, and preventing their re-trapping, thereby leading to an enhanced conductivity in the MoS_2_ channel under NIR illumination. This mechanism is described pictorially in Fig. 4.

While further studies are needed to fully understand, and thereby optimize, the NIR photocurrent response, certain possibilities can be ruled out at this stage. Unlike for previous studies of MoS_2_ sensitization by QDs^9^ and films of organic dye molecules^8^, it seems unlikely that charge transfer plays a role in the photocurrent response since the LUMO level of **2** is almost the same as that reported for MoS_2_^30^ (*c*.*f*. Table 1) and would be lower in the case of [**2**]^−^. Furthermore, in a control experiment conducted by drop casting of a film of **1b** on the surface of a MoS_2_ device, only negligible photocurrent could be detected in the NIR, despite its higher extinction coefficient and significantly higher surface coverage (Fig. S10, ESI). Given the similarity of the frontier orbital energy levels between **1b** and **2** (*c*.*f*. Table 1), it can be reasoned that charge transfer does not play a role in the photocurrent mechanism. Likewise, if a photothermal mechanism were underlying the photocurrent response in the NIR, it could reasonably be expected that the stronger NIR absorption of a drop cast film of **1b** on a MoS_2_ device would lead to an enhanced NIR photoresponse, compared to that of MoS_2_-**2** (for which the NIR absorption could not be clearly observed, *c*.*f*. Fig. 2c). However, this is not the case and thus it is possible to conclude that a photothermal effect does not significantly contribute to the NIR photoresponse of MoS_2_-**2**.

## Conclusions

In this study, the sensitization of MoS_2_ films *via* covalent attachment of a heteroleptic NiDT complex to sulfur vacancies in the MoS_2_ surface is demonstrated. The sensitizer is shown to extend the range of light detection for MoS_2_ phototransistors up to 1500 nm, thereby effectively covering the 2^nd^ telecommunications window and, despite a low surface coverage (not directly detectable under absorption spectroscopy), and weak solvatochromism, the NIR sensitization yields a clear, stable and reproducible photocurrent response. Further study will look at optimizing the molecular sensitizer towards stronger dipole change under illumination, as well as optimizing the sensitization method to achieve enhanced sensitizer response without compromising the intrinsic performance of the MoS_2_. The use of passive dielectrics and top contact architectures are expected to greatly improve the general optoelectronic response of such sensitized devices, allowing a more detailed analysis of especially the relative carrier dynamics following MoS_2_ and sensitizer stimulation. Furthermore, fabricating devices with an appropriately chosen interlayer (*e*.*g*. TiO_2_) between the MoS_2_ and the sensitizer molecules^10^ might help protect the MoS_2_ channel from serendipitous adsorbates, which can adversely affect the (opto)electronic properties of the devices, as well as to increase dye loading. The resultant devices would also help to clarify the mechanism of photocurrent enhancement - whether the dye molecule is acting on the localized traps or on the electronic bands of the MoS_2_. It should be noted that the proposed mechanism of conductivity enhancement under sensitizer stimulation is not necessarily limited to photodetection, but would, in principle, also be applicable to technologies, such as optical memories (through the use of photochromic sensitizers) - as long as the sensitizer stimulation results in a local change to dielectric environment close to the MoS_2_ surface.

## Experimental

The synthesis of **1a** and **2** is described in detail in the ESI, and follows a typical route for NiDTs^31^. MoS_2_ was purchased from 2DSemiconductors as a single batch as continuous monolayer films on 10 × 10 mm^2^ sapphire wafers. Dye-sensitization was carried out based on the method of Sim *et al*.^13^ whereby the (transferred) samples were annealed for 1 hr at 250 °C in a N_2_ filled glovebox. Following rapid cooling, the samples were soaked in a 0.5 mM solution of **2** in DCM and stored in the dark for >48 hrs. The samples were extensively rinsed with fresh DCM, and were then annealed on a hotplate at 50 °C for 10 mins.

UV/Vis/NIR spectroscopy (Perkin Elmer Lambda950) on solutions and thin films were measured in transmission mode. Cyclic voltammetry (Keithley 2450EC) was performed in a three electrode configuration (reference: Ag/Ag^+^; working: glassy carbon; counter: platinum wire) on *ca*. 1 mM analyte solution in DCM using 0.1 M TBABF_4_ (TBA = tetrabutylammonium) as supporting electrolyte.

For (opto)electronic measurements, BGBC transistor substrates comprising Si/SiO_2_ (300 nm) with ultra-thin Au source drain electrodes using a mixed self-assembled monolayer (SAM) of (3-Aminopropyl)trimethoxysilane/(3-Mercaptopropyl)trimethoxysilane as an adhesion layer^22^. Au source drain electrodes (*d* = 9 nm) were deposited under physical vapour deposition at a rate of 0.3 Ås^−1^ using a shadow mask to define channel dimensions *L* = 30 μm/*W* = 1 mm. The substrates were briefly subjected to oxygen plasma immediately prior to film transfer to remove exposed SAM. MoS_2_ transfer was achieved by surface-energy-assisted transfer using polystyrene as a support, following the method of Gurarslan *et al*.^21^. In short, the as-received MoS_2_ was spin coated with a solution of polystyrene (PS) (M_w_ = 280 kg/mol, 90 mg/ml in toluene) at 2000 rpm and baked at 90 °C for 15 mins. The film was divided into 4 by scratching with a razor blade. A droplet of water was then applied to release the divided MoS_2_/PS films from the substrate. The floating films were aligned over the electrode arrays of different substrates and the excess water was removed with a lint-free tissue. After air-drying for 30 mins, the devices were annealed for 1 hr at 80 °C, and further at 150 °C for 30 mins. Polystyrene was removed by rinsing with toluene and the pixel was isolated using a cotton bud. For transistor devices, dye-sensitization was performed after transfer, exactly as described above. Note: the conductivity of the bare ultra-thin electrodes was observed to diminish with aging, due to aggregation of the Au in the film. This process was accelerated by annealing, but was averted when the film was covered by *e*.*g*. MoS_2_. Therefore, in the used devices, the ultra-thin region only extended to the source/drain contacts, which were fabricated immediately before MoS_2_ transfer which completely covered this region. The traces and contact pads comprised 70 nm Au, with 5 nm Cr as adhesion layer, which provided robust external contacts. The electrical properties of such electrodes were further assessed relative to conventional BGBC devices (70 nm Au/5 nm Cr) using P3HT as semiconductor and essentially showed no statistical difference between the two electrode architectures^32^.

Action spectra measurements were performed by a lock-in method at 263 Hz, as reported previously^33^ over a wavelength range 350–1700 nm, as further described in the ESI. Phototransistor characterization (Keithley 2636B/LabVIEW) was performed in the dark or under illumination from a fibre-coupled LED (*λ*_max_ = 640 nm or 1310 nm, *P*_max_ = 260 μWcm^−2^ set for both).

### Supporting information

Further experimental details, as well as additional experimental data on synthesis, characterization and device testing are available in the Electronic Supporting Information. This material is available free of charge *via* the Internet at.

## Supplementary information

Supporting Information

## Data Availability

The datasets generated during and/or analysed during the current study are available from the corresponding author on reasonable request.
