# Supplementary material for: Potential modulations in flatland: near-infrared sensitization of MoS2 phototransistors by a solvatochromic dye directly tethered to sulfur vacancies
Source: Sci Rep. 2019 Nov 13;9:16682. doi: 10.1038/s41598-019-53186-2 (PMC6853947; doi:10.1038/s41598-019-53186-2)
Supplement: Supplementary file 1 — Supporting Information [file 41598_2019_53186_MOESM1_ESM.pdf]

Electronic Supporting Information (ESI)

Potential modulations in flatland: near-infrared sensitization of MoS_2 phototransistors by a solvatochromic dye directly tethered to sulfur vacancies

Simon Dalgleish[1,2]*, Louisa Reissig[3], Yoshiaki Shuku[1], Giovanni Ligorio[2], Kunio Awaga[1], Emil J. W. List-Kratochvil[2,4]

[1] *Department of Chemistry and IRCCS, Nagoya University, Furo-cho, Chikusa, 464-8602 Nagoya, Japan*

[2] *Institut für Physik, Institut für Chemie & IRIS Adlershof, Humboldt-Universität zu Berlin, Brook-Taylor-Str. 6, 12489 Berlin, Germany*

[3] *Institute of Experimental Physics, Freie Universität Berlin, Arnimallee 14, 14195 Berlin, Germany*

[4] *Helmholtz-Zentrum Berlin für Materialien und Energie GmbH, Brook-Taylor-Str. 6, 12489 Berlin, Germany*

CONTENTS:

1) **Materials and Methods - Page 2**
   - Synthetic Route to Ni(C_4dtpdt)(*i*Pr_2timdt) - Page 2
   - Molecular Characterization - Page 7
   - Film Characterization - Page 9
   - Device Fabrication and Testing - Page 10
2) **Supporting Data - Page 12**
   - Molecular Characterization - Page 12
   - Film Characterization - Page 19
   - Device Fabrication and Testing - Page 22
3) **References - Page 25**

MATERIALS AND METHODS

Synthetic Route to Ni(C_4dtpdt)(iPr$_2$timdt) (2)

All reagents and solvents were used as received, unless otherwise stated. Ni(iPr$_2$timdt)$_2$ was synthesized following the method of Bigoli *et al.*[1]

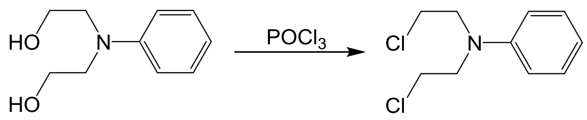

<u>Step 1:</u>[2] To phosphorous oxychloride (52 ml, 0.552 mol), cooled to 0°C in an ice bath, was slowly added powdered *N,N*-bis(hydroxyethyl)aniline (50.0 g, 0.276 mol). The mixture was slowly warmed to 100°C in an oil bath (<u>Caution</u>: large quantities of HCl gas evolved). The reaction was stirred at this temperature for 1 hr, cooled and diluted with benzene (250 ml). This solution was then poured into 125 g of crushed ice and the layers separated. The aqueous layer was extracted with 3 x 40 ml of benzene and the combined organics were dried over Na_2SO_4. Removal of the solvent left a reddish oil which was crystallized from hot methanol, to give *N,N*-bis(chloroethyl)aniline as colorless crystals (55.0 g, 91%). δ_H (400 MHz; $CDCl_3$) 3.64 (t, 4H, J = 6.7 Hz), 3.73 (t, 4H, J = 6.7 Hz), 6.7 (d, 2H, J = 8.8 Hz), 6.78 (t, 1H, J = 7.3), 7.27 (dd, 2H, J = 8.8, 7.3 Hz).

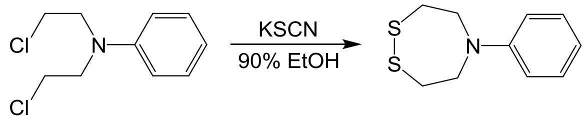

<u>Step 2:</u>[3] *N,N*-bis(chloroethyl)aniline (52.1 g, 0.239 mol) and KSCN (465 g, 4.78 mol) were suspended in 1.2 L EtOH/H_2O (9/1) and the reaction mixture was taken to vigorous reflux with stirring. Upon heating, the suspension dissolved, and formed a new precipitate after about 10 mins. After 36 hrs, the reaction, which had become a dark brown color, was cooled to room temperature, and the solvent removed under reduced pressure. The crude product was dispersed in DI H_2O (600 ml) and filtered, yielding a brown powder. This was loaded onto a silica plug and was eluted with $CHCl_3$ until no aromatics were observed by TLC. The solvent was removed, leaving a yellow oil that was triturated with MeOH for 24 hrs. Following filtration, 5-Phenyl-[1,2,5]-dithiazepane was obtained as a white crystalline

precipitate (36.5 g, 72%). δ_H (400 MHz; $CDCl_3$) 3.10 (t, 4H, J = 5.6 Hz), 3.97 (t, 4H, J = 5.6 Hz), 6.64 (d, 2H, J = 8.8 Hz), 6.70 (t, 1H, J = 7.3), 7.23 (dd, 2H, J = 8.8, 7.3 Hz).

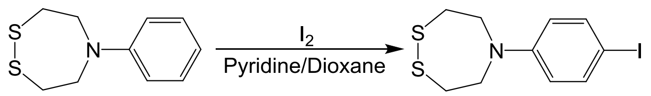

Step 3:[4] To a stirred solution of 5-Phenyl-[1,2,5]-dithiazepane (9.40 g, 44.5 mmol) in pyridine/1,4-dioxane (1:1, total 120 ml), cooled to 0°C in an ice bath, was added iodine (16.9 g, 66.7 mmol), in a single portion, and the reaction stirred at this temperature for 1hr. The ice bath was removed and a further portion of iodine (5.64 g, 22.2 mmol) was added and the reaction was stirred for a further hour. After this time, no trace of starting material could be seen by TLC and thus a sat. solution of sodium thiosulfate was added until the reaction mixture decolored. The reaction mixture was extracted with dichloromethane (DCM) (1 L) and was washed with DI H_2O (750 ml), and the organic phase dried over Na_2SO_4 and concentrated under reduced pressure, leaving a green oil. This was loaded onto a short silica plug and eluted with 1:1 DCM/hexane until no organics could be seen by TLC. The solvent was removed under reduced pressure, leaving a yellow oil. This was crystallized from hot methanol and triturated in MeOH for 24 hrs. Following filtration, 4-Iodo-5-phenyl-[1,2,5]-dithiazepane was obtained as a white crystalline precipitate (12.3 g, 82%). δ_H (400 MHz; $CDCl_3$) 3.06 (t, 4H, J = 5.6 Hz), 3.93 (t, 4H, J = 5.6 Hz), 6.41 (d, 2H, J = 9.1 Hz), 7.46 (d, 2H, J . 9.1 Hz).

Note: 4-Iodo-5-phenyl-[1,2,5]-dithiazepane decomposes rapidly upon storage under normal laboratory conditions (takes on a brown color and yields poorly in subsequent steps). The rate of decomposition can be slowed by storing in a refrigerator under an inert atmosphere. Alternatively, degraded product can be purified prior to use by passing through a silica plug using DCM/hexane (1:1) as eluent.

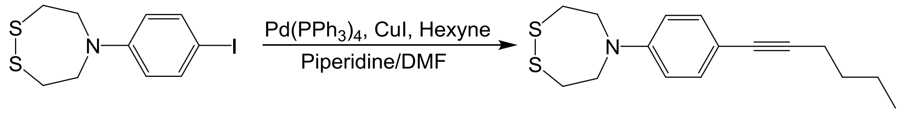

Step 4: $Pd(PPh_3)_4$ (5.00 g, 4.33 mmol) and CuI (634 mg, 3.33 mmol) were added to an oven-dried 500 ml 3-necked round bottomed flask, equipped with a 200 ml dropping funnel, pre-purged with argon. The apparatus was further evacuated and purged with argon 3 times, and previously degassed piperidine (200 ml) was added via cannula, and the reaction mixture further degassed with bubbling argon. To the dropping funnel was added 4-iodo-5-phenyl-[1,2,5]-dithiazepane (11.22 g, 33.3 mmol) and dry degassed DMF (55 ml). This mixture was further degassed with bubbling argon for 20 mins, whereupon 1-hexyne (72 ml, 629 mmol) was added and the mixture further degassed for 10mins. The contents of the dropping funnel were added quickly to the catalyst mixture, which took on a dark brown color over 5 mins with moderate exotherm. When the exotherm subsided, a white precipitate started to form, and the reaction mixture became visibly paler. The reaction was warmed to 55°C, whereupon the precipitate dissolved and the reaction mixture became dark once again. After a further 4 hrs, the reaction mixture became paler once again (with no precipitate) and the heat was removed, and the reaction stirred overnight. The solvent was removed under reduced pressure, and the crude product was dissolved in Et_2O (800 ml), washed with DI H_2O (3 x 400 ml) and sat. brine (300 ml). The organic phase was dried over Na_2SO_4, and the solvent removed under reduced pressure. The crude product was loaded onto a silica column and eluted with 1:2 Et_2O/hexane, collecting the second band. Residual Ph_3PO, that had survived the workup was observed in the initial product, but could be removed by repeated (2 times) trituration in cold MeOH, yielding pure 4-hexynyl-5-phenyl-[1,2,5]-dithiazepane as a pale yellow powder (6.62 g, 68%). δ_H (400 MHz; $CDCl_3$) δ_H (400 MHz; $CDCl_3$) 0.94 (t, 3H, J = 7.3 Hz), 1.42-1.52 (m, 2H), 1.54-1.61 (m, 2H), 2.39 (t, 2H, J = 7.0 Hz), 3.07 (t, 4H, J = 5.6 Hz), 3.95 (t, 4H, J = 5.6 Hz), 6.53 (d, 2H, J = 8.9 Hz), 7.27 (d, 2H, J . 8.9 Hz).

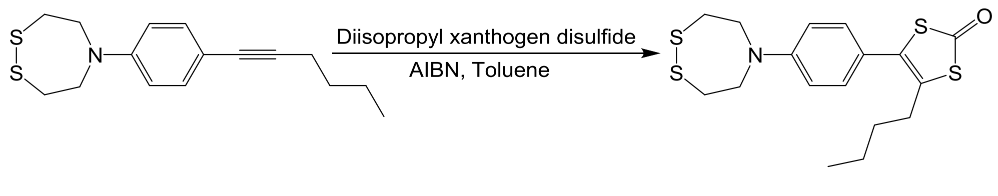

Step 5:[5] To an oven dried flask, equipped with condenser, was added 4-hexynyl-5-phenyl-[1,2,5]-dithiazepane (6.60 g, 22.6 mmol), diisopropyl xanthogen disulfide (9.80 g, 36.2 mmol) and 2,2'-Azobis(2-methylpropionitrile) (2.23 g, 13.6 mmol) and the flask was purged with

nitrogen. Toluene (3.3 ml) was added and the reaction mixture was warmed to 100°C overnight (<u>Caution</u>: at this scale, extensive degassing occurs at ~80°C). The reaction mixture was cooled and the toluene was removed under reduced pressure. The crude product was purified by column chromatography (DCM/hexane 4:1), followed by trituration in cold MeOH to remove residual *O*-isopropyl-*S*-isobutyronitrile dithiocarbonate. Following filtration, pure 4-butyl-5-phenyl-[1,2,5]-dithiazepane-[1,3]dithiol-2-one was obtained as a straw colored powder (7.90 g = 91%). δ_H (400 MHz; $CDCl_3$) 0.87 (t, 3H, J = 7.3 Hz), 1.27-1.38 (m, 2H), 1.50-1.58 (m, 2H), 2.59 (t, 2H, J = 7.7 Hz), 3.10 (t, 4H, J = 5.6 Hz), 3.98 (t, 4H, J = 5.6 Hz), 6.63 (d, 2H, J = 8.8 Hz), 7.19 (d, 2H, J = 8.8 Hz); FAB-MS *m/z* = 383.0514 $[M]^+$; calc for $C_{17}H_{21}NOS_4$: 383.0506.

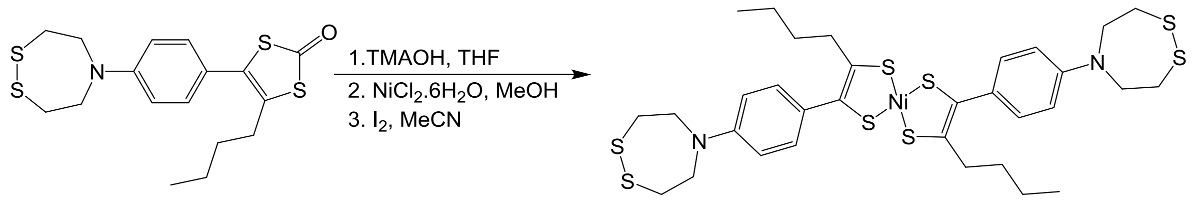

<u>Step 6:</u> To a stirred solution of 4-butyl-5-phenyl-[1,2,5]-dithiazepane-[1,3]dithiol-2-one (500 mg, 1.30 mmol) in dry, degassed tetrahydrofuran (THF) (20 ml) under nitrogen was added a previously degassed 25 wt.% solution of tetramethylammonium hydroxide in MeOH (1.1 ml, 2.67 mmol). The reaction mixture was stirred for 5 mins, whereupon a degassed solution of $NiCl_2{\cdot}6H_2O$ (1.65 mg, 0.696 mmol) in MeOH (2 ml) was added dropwise, causing an instant color change to deep red. After a further 30 mins of stirring, I_2 (331 mg, 1.30 mmol) in MeCN (2 ml) was added dropwise. The reaction was stirred for a further 30 mins, over which time the reaction mixture turned deep green with the formation of a green precipitate. EtOH (50 ml) was added, and the reaction mixture was concentrated to half the initial volume under reduced pressure and then filtered to yield **1a** as an olive green precipitate. The crude product was passed over a short silica plug, eluting with DCM, and the target complex was isolated by recrystallization from DCM/EtOH to yield a green powder (263 mg, 52%). δ_H (400 MHz; $CDCl_3$) 0.88 (t, 6H, J = 7.4 Hz), 1.30-1.41 (m, 4H), 1.81-1.90 (m, 4H), 2.92 (t, 4H, J = 7.8 Hz), 3.13 (t, 8H, J = 5.5 Hz), 4.02 (t, 8H, J = 5.5 Hz), 6.69 (d, 4H, J = 8.8 Hz), 7.43 (d, 4H, J = 8.8 Hz); FAB-MS *m/z* = 768.0467 $[M]^+$; calc for $C_{32}H_{42}N_2NiS_8$: 768.0439.

Note: The timing of additions is critical to obtaining a reasonable yield. Delayed addition of TMAOH or I_2, as well as the use of NaOMe in MeOH as base (which is slower due to the poor solubility of 4-Butyl-5-phenyl-[1,2,5]-dithiazepane-[1,3]dithiol-2-one in MeOH), resulted in significantly reduced yields. It is likely that the disulfide bond of the dithiazapane unit is vulnerable to reduction by the dithiolate and/or the dianionic complex, which then interferes with complex formation.

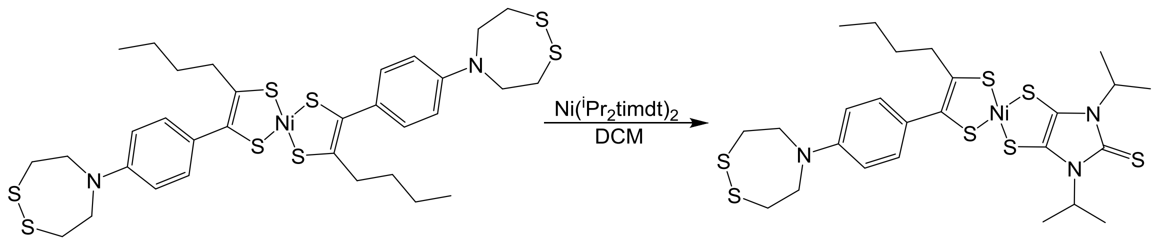

<u>Step 7:</u> To complex **1a** (282 mg, 0.367 mmol) and Ni(*i*Pr$_2$timdt)$_2$ (**1b**) (202 mg, 0.367 mmol) was added dry, degassed DCM (100 ml) and the mixture taken to reflux for 24 hrs. The progress of the reaction was monitored by TLC. The solvent was removed under reduced pressure and the crude product mixture was purified by column chromatography using CS_2/DCM (2:1) as eluent. Following concentration under reduced pressure, the target complex was recrystallized from DCM/EtOH to yield **2** as a dark green powder (98 mg, 20%). δ_H (400 MHz; $CDCl_3$) 0.89 (t, 3H, J = 7.3 Hz), 1.30-1.41 (m, 2H), 1.75 (d, 12H, J = 7.0 Hz), 1.78-1.87 (m, 2H), 2.99 (t, 2H, J = 7.8 Hz), 3.13 (t, 4H, J = 5.5 Hz), 4.02 (t, 4H, J = 5.5 Hz), 5.57 (sep, 2H, J = 7.0 Hz), 6.69 (d, 2H, J = 8.8 Hz), 7.39 (d, 2H, J = 8.8 Hz); FAB-MS *m/z* = 659.0229 $[M]^+$; calc for $C_{25}H_{35}N_3NiS_7$: 659.0218.

Molecular Characterization

Cyclic voltammetry was performed at room temperature on a *ca.* 1 mM analyte solution in dry, degassed DCM using 0.1 M $TBABF_4$ as supporting electrolyte. A three electrode configuration was used with Ag/Ag^+ serving as the reference electrode and a glassy carbon disk (7 mm^2) and platinum wire serving as the working and counter electrodes, respectively. All measurements were calibrated and reported against the Ferrocene/Ferrocenium (Fc/Fc^+) redox couple, which was added to the cell following each data set. Each process was measured over a range of scan rates (*v)* between 10-100 mVs^{-1} and electrochemical reversibility was assigned based on a linear relation of peak current i_p vs. $v^{1/2}$ and an invariant peak-to-peak ($\Delta E^{a/c}$) separation with scan rate. In the case of irreversible processes, and for the purposes of energy level estimation, the $E_{1/2}$ value quoted is based on the relative shift in the onset of oxidation (reduction), compared to that of Fc/Fc^+. All measurements were performed using a Keithley 2450-EC source meter.

Solution UV/Vis/NIR measurements were recorded in various solvents using a quartz cell of path length 10 mm on a Perkin-Elmer Lambda 950 spectrophotometer. Spectroelectrochemical measurements were recorded in a quartz cell of path length 0.5 mm on 0.5 mM solution of **2** in dry degassed DCM using 0.1 M $TBABF_4$ as supporting electrolyte in a three electrode cell. The working electrode was a Pt/Rh gauze, the reference electrode was Ag/Ag^+ and the counter electrode was a platinum wire.

Crystals of **1a** and **2**, suitable for analysis, were grown by slow mixing of a DCM solution of **2** with hexane. The X-ray diffraction data analyses were collected on a Rigaku AFC-10 instrument equipped with a Saturn 70 CCD detector by using graphite-monochromated Mo Kα radiation (λ = 0.71075 Å) under a cold nitrogen stream. The frame data were integrated and corrected for absorption with the Rigaku/MSC Crystal Clear package.[6] The structures were solved by direct method (SIR92)[7] and standard difference map techniques, and were refined with full-matrix least-square procedures on F^2. All calculations were performed using the crystallographic software package, Crystal Structure,[8] except for refinements, which were performed using SHELXL2016/4.[9] Anisotropic refinement was applied to all non-hydrogen atoms. All hydrogen atoms were placed at calculated positions and refined using a riding model. The electron densities of crystal solvent in the cavity of **2**'s crystal structure were flattened, using the SQUEEZE subroutine of PLATON,[10] and only the

molecules of **2** were refined. Two different arrangement of 1,2,5-dithiazepane ring were observed as positional disorders of the sulfur atoms in **1b** (S3 and S4) and **2** (S3 and S4). They were treated by disorder models and the occupancies of major arrangement were 0.859(2) in **1b** and 0.833(6) in **2**. CCDC 1887344 and 1887345 contain the supplementary crystallographic data for this paper. This data can be obtained free of charge from The Cambridge Crystallographic Data Centre via www.ccdc.cam.ac.uk/data_request/cif.

Film Characterization

Dye-sensitization was carried out based on the method of Sim *et al*,[11] as reported in the manuscript. Thin films of **2** were fabricated by spin coating from dichloroethane to yield a homogeneous film of 50 nm thickness (*c.f.* Fig. 2c), as measured by surface profilometry (Dektak XT, scan speed = 4 µm/s, stylus force = 1 mg).

Thin film absorption measurements of MoS_2 (as received and processed) were recorded on the 10×10 mm^2 c-cut sapphire substrates on which they were supplied (2DSemiconductors). In all cases, the spectrum of a clean sapphire substrate was used as background. Note: all spectra of as received MoS_2 films showed a slight positive y-offset and noise around 1150 nm, which were not apparent after further processing (*c.f.* Fig. 2c, right inset). It is likely that the as received samples retained some residue from their packaging and/or processing that decreased transmittance/increased reflectance in this region. This effect was reduced upon further processing (heating to 250°C, and soaking in DCM – common to MoS_2-C, MoS_2-**1b** and MoS_2-**2**). Thin film absorption measurements of **2** were measured on 10×10 mm^2 quartz substrates. The substrates were fixed perpendicular to the incident beam with the beam profile reduced to ensure all light passed through the sample. Spectra were measured in transmission mode.

Atomic force microscopy (AFM) was using to characterize the surface of the monolayer films, and was performed on a Bruker Dimension Icon AFM system with ScanAsyst Probes. Samples were scanned in Peak Force Tapping™ mode (512 lines, scan speed ≤ 0.5 Hz). Images were analyzed using Gwyddion software (v2.52).[12] When necessary the images were level corrected. The RMS surface roughness (R_z) was determined from at least 6 cross-sections.

Device Fabrication and Testing

For (opto)electronic measurements, a bottom-gate bottom-contact (BGBC) device architecture was used based on Si/SiO_2 substrates (d_{ox} = 300 nm). The source/drain electrodes were either ultra-thin Au electrodes (molecular adhesion layer) with channel dimension L = 30 µm/W = 1 mm, as described in the manuscript, or were interdigitated array electrodes (IDE) (80 nm Au/5 nm Ti), with channel dimensions L = 5 µm/W = 32 cm, prepared by standard photolithographic methods. IDE substrates were cleaned by sequential mild bath ultrasonnication in acetone, propan-2-ol, DI H_2O and blown dry with nitrogen. The substrates were further briefly subjected to oxygen plasma immediately prior to film transfer. MoS_2 transfer was achieved following the method of Gurarslan *et al.*[13] (see Fig. S7), as described in the manuscript.

Optoelectronic characterization was performed under inert and dark conditions (unless otherwise stated) on a Keithley2636B SourceMeter controlled via a home-written Labview program.

Two different optical configurations were used in this study:

(1) Fixed wavelength measurements:[2,3] Light from an LED (λ_{max} = 639 nm or 1310 nm) was directed onto a collimator via an optical fiber, resulting in a 7 mm homogeneous broad beam. The light power was adjusted before each experiment by a home-built driver unit (circuit diagram available upon request) up to a maximum power of 260 µWcm^{-1}.

(2) Wavelength dependent measurements (Action spectrum):[3,4] The devices were illuminated by a tungsten/halogen light source (Spectral Products ASBN-W 150F-L) attached to a dual grating monochromator (Digikröm CM110, grating 1: 1200 Grv/mm (blaze 300 nm), grating 2 600 Grv/mm (blaze 1200 nm), slits 1.2 mm (Vis) and 0.6 mm (NIR), resolution 8 – 10 nm), and modulated by a chopper. Harmonics in the light source were removed by high-pass filters. The photocurrent signals were pre-amplified using a low noise transimpedance amplifier (Femto, DLPCA 200) and extracted using a lock-in amplifier (Perkin Elmer 7265 DSP). Digital to analogue conversion was performed using a digital multimeter (Keithley 2110) and the signal was recorded on a computer. The setup was controlled by a home-written LabVIEW program, scanning the wavelength range

between 350 to 1700 nm in both directions to confirm signal shape and stability. The responsivity was calculated by dividing by the wavelength-dependent light power (see Fig. SX1) to obtain the responsivity spectra. Where indicated, the NIR region was measured using 1.2 mm slits (resolution 20 nm) to confirm the presence/absence of a peak at a higher light intensity. It should be noted that the overall shape of the NIR peak in the MoS_2-**2** devices was unaffected by the slits used.

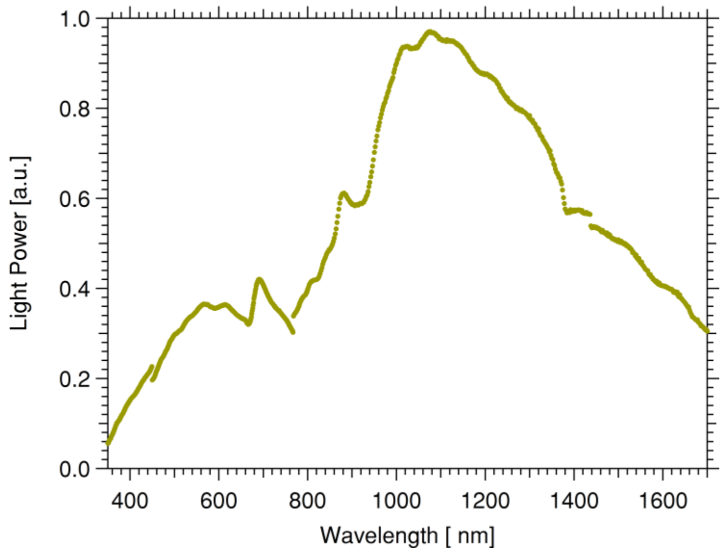

Figure SX1: Light power spectrum used for the action spectrum measurement.

SUPPORTING DATA

Molecular Characterization

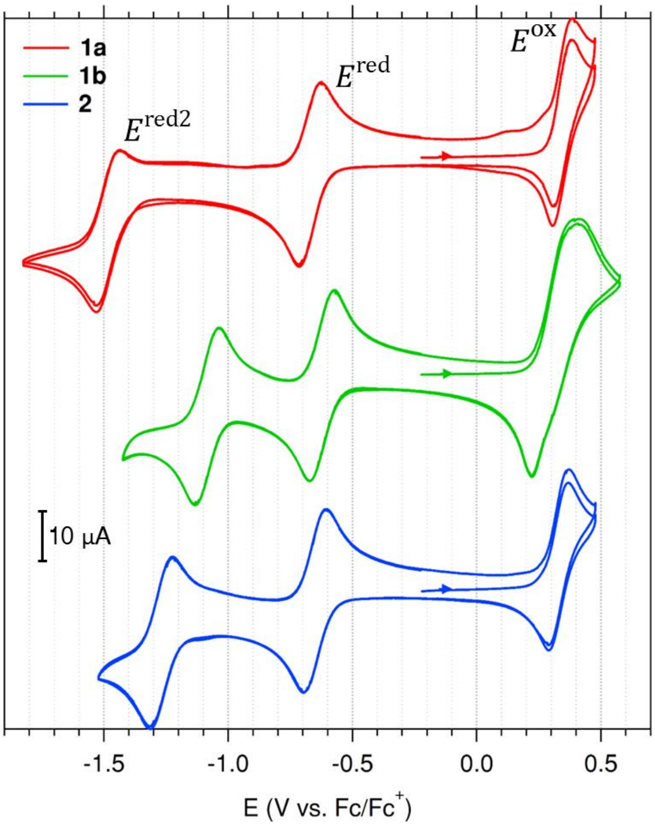

Figure S1: Electrochemical data for **1a**, **1b** and **2**, showing the three redox process, corresponding to the first and second reduction processes (E^{red} and E^{red2}, respectively) and the oxidation process (E^{ox}) of the complexes. Each voltammogram shows the first and second cycle, with the initial direction of scan indicated by arrowhead.

Further comment: It is interesting to note that while **2** shows two electrochemically reversible reduction processes in its CV, which are a common trait of neutral NiDTs,[14] the second reduction process of **1a** is not fully reversible, and instead showed EC-like character. This might indicate that the disulfide bond in the dithiazapane unit is vulnerable in this highly reduced state (either via a monomolecular or bimolecular process), the opening of which might lead to decomposition via attack on dithiolene core. This latent reactivity of the dithiazapane unit might also explain why the synthesis of complex **1a** was observed to be time-sensitive (see above discussion on the synthesis of **1a**) and why the ligand scrambling

reactions involving a change in oxidation state failed for **1a** (such as with salts of $[Ni(mnt)_2]^{2-}$, $[Ni(dmit)_2]^{2-}$) when they have all been achieved in our lab using the structurally and electronically analogous *bis*-(4-dimethylaminodithiobenzil)-Ni(II) (BDN).

Table S1: Solvatochromic data for **2** showing peak absorption wavelength (λ_{max}) and wavenumber ($\bar{\nu}$) compared to values of macroscopic solvent parameters:[15] refractive index (*N*) and dipole moment (μ), as well as Kamlet & Taft's π* empirical values of solvent polarity.[16] ‡ Denotes spontaneous reduction of complex – reported value for peak of neutral complex uncorrected for skewing.

Name	#	*N*	*μ*	*π**	λ_{MAX} (nm)	$\bar{\nu}$ (cm^{-1})
Carbon Disulfide	**1**	1.626	0	0.514	962	10395.01
o-Dichlorobenzene	**2**	1.552	5.57	-	950	10526.32
Chlorobenzene	**3**	1.525	5.14	0.709	945	10582.01
Toluene	**4**	1.497	1.43	0.535	938	10660.98
p-Xylene	**5**	1.496	0	0.426	936	10683.76
Dimethylsulfoxide	**6**	1.478	13	1	*959*‡	*10427.53*‡
Cyclohexane	**7**	1.476	0	0	917	10905.13
Carbon tetrachloride	**8**	1.46	0	0.294	930	10752.69
Chloroform	**9**	1.445	3.84	0.76	938	10660.98
1,2-Dichloroethane	**10**	1.445	6.2	0.807	940	10638.3
N,*N*-Dimethylformamide	**11**	1.431	12.88	0.875	*949*‡	*10537.41*‡
Dichloromethane	**12**	1.424	5.17	0.802	933	10718.11
1,4-Dioxane	**13**	1.422	1.5	0.553	933	10718.11
Tetrahydrofuran	**14**	1.407	5.84	0.576	936	10683.76
1,2-Dimethoxyethane	**15**	1.38	5.7	-	935	10695.19
Ethyl acetate	**16**	1.372	6.27	0.545	927	10787.49
Acetone	**17**	1.359	9.54	0.683	931	10741.14
Acetonitrile	**18**	1.344	11.48	0.713	927	10787.49

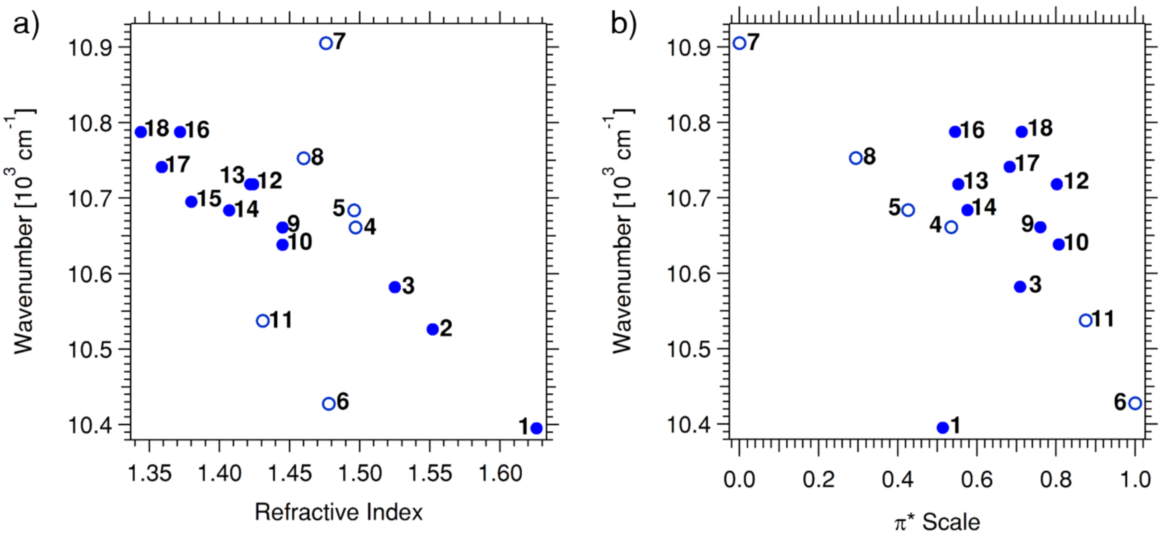

Figure S2: Solvatochromism of **2** showing the energy of the $\pi \rightarrow \pi^*$ transition in units of cm^{-1}, plotted against (a) the refractive index of the solvent[15] (significant outliers plotted as open circles), and (b) the π^* scale of Kamlet & Taft[16] (outliers from (a) are plotted as open circles). In both cases, solvent numbering scheme corresponds to that in Table S1.

Note: In the case of DMF and DMSO, the spectrum shows the presence of two peaks. With time, the relative intensity of the two peaks changed (red peak↑, blue peak↓). The position of the emergent red peak is similar to that of the reduced complex measured by spectroelectrochemistry (*c.f* Fig. S3) and thus it seems that such solvents (both of high donor number) causes the spontaneous reduction of **2**. Such solvent-induced redox change has been previously reported for other NiDTs, including for **1b**.[17]

Further comment: While **2** shows a reasonable correlation (negative solvatochromism) with the solvent refractive index, significant outliers are found in the case of cyclohexane, DMF and DMSO. When the data are re-plotted against the empirical π* scale of Kamlet & Taft,[16] (which was developed to correlate solvatochromic effects on π→π* electronic transitions) these outlying solvents show the strongest correlation, with the direction of solvatochromism reversed (positive solvatochromism). NiDTs that can be characterized as π-delocalized, such as homoleptic complexes and those showing weak intermolecular charge transfer, generally show weak positive solvatochromism,[18] whereas the π-localized "push-pull" NiDTs can show strong negative solvatochromism due to presence of a large ground

state dipole, which is reduced upon photoexcitation. However, in the case of **1b**, weak negative solvatochromism has previously been reported,[17] ($\Delta\lambda$ = 12 nm (122 cm^{-1}) between CS_2 and MeCN) despite its symmetric structure and, thus, absence of a ground state dipole. In this case, the nature of the solvatochromism shown by the molecule is likely derived from specific solvent interaction with the molecule rather than by the macroscopic solvent parameters. By analogy, for **2**, it is not clear whether the predominantly weak negative solvatochromism is evidence for intermolecular charge transfer, or rather its susceptibility to interact with certain solvents. However, even in the extreme case of equal ligand contribution to the $\pi \rightarrow \pi^*$, photoexcitation would still be expected to move electron density from the C_4dtpdt towards the core of the molecule, and thus away from any surface to which the molecule is bound.

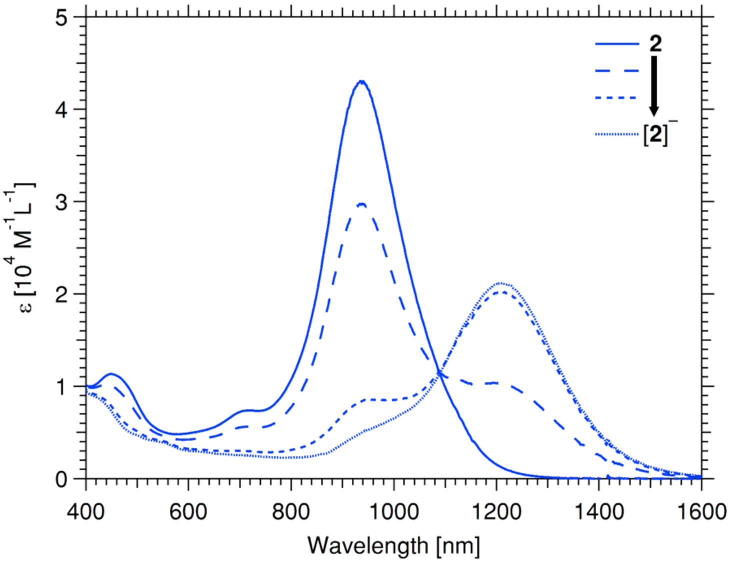

Figure S3: Evolution of the absorption spectrum of **2** under electrochemical reduction.

Table S2: Crystallographic data for Ni(C_4dtpdt)$_2$ (**1a**) and Ni(C_4dtpdt)(*i*Pr_2timdt) (**2**).

**Complex**	Ni(C_4dtpdt)$_2$ (**1a**)	Ni(C_4dtpdt)(*i*Pr_2timdt) (**2**)
**Formula**	$C_{32}H_{42}N_2NiS_8$	$C_{25}H_{35}N_3NiS_7$
**Formula weight /g mol^{-1}**	769.88	690.69
**Dimension / mm^3**	0.28 x 0.08 x0.08	0.07 x 0.07 x 0.01
***T* / K**	123	123
**Crystal system**	Monoclinic	Monoclinic
**Space group**	*P*2/*c* (#13)	$P2_1/c$ (#14)
***a* / Å**	16.236(3)	19.448(3)
***b* / Å**	10.4776(18)	11.8135(18)
***c* / Å**	21.635(4)	29.026(5)
***β* / °**	111.925(2)	104.989(2)
***V* / Å^3**	3414.2(11)	6441.7(18)
Z	4	8
**D_{calc} / g cm^{-3}**	1.498	1.362
***μ*(Mo Kα) / cm^{-1}**	10.847	10.757
***F*(000)**	1616.00	2768.00
**$2\vartheta_{max}$ / °**	55.0	54.9
**Reflections collected**	26123	50719
**Unique reflections (R_{int})**	7663 (0.0328)	14644 (0.0785)
**Number of parameters**	409	678
**Final R_1 [$I > 2\sigma(I)$][a]**	0.0531	0.0773
**wR_2[b]**	0.1379	0.1982
**Goodness of fit**	1.092	1.053
[a] $R_1 = \Sigma\|\|F_o\|-\|F_c\|\|/\Sigma\|F_o\|$		
[b] $wR_2 = [\Sigma\{w(F_o^2-F_c^2)^2\}/\Sigma w(F_o^2)^2]^{1/2}$		

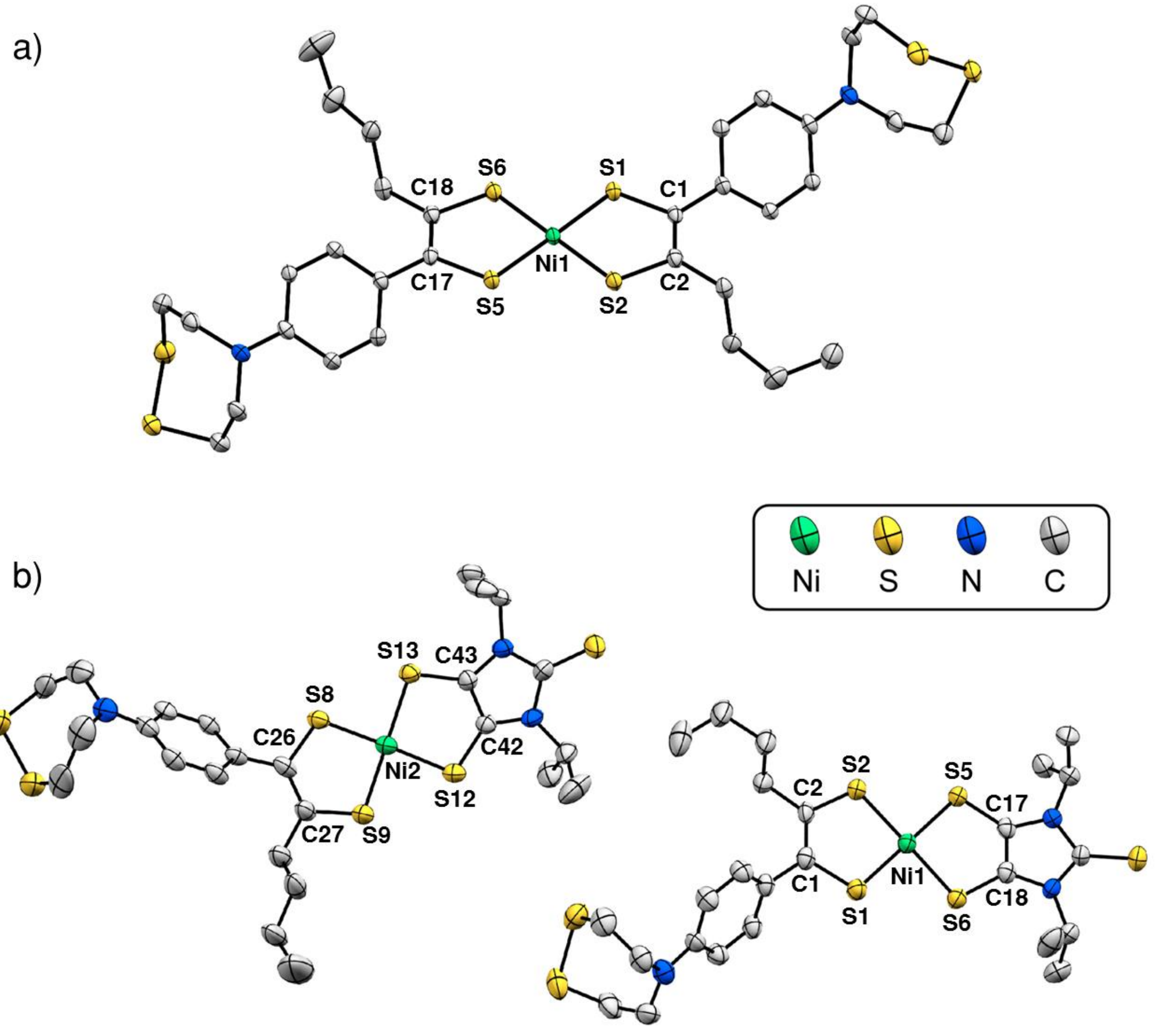

Figure S4: Molecular structures and atom labeling scheme for core atoms (a) **1a**, and (b) **2**. Ellipsoids set at 50% probability. Selected bond lengths (Å) and (torsion) angles (°): (a) Ni1-S1: 2.1252(7), Ni1-S2: 2.1186(9), Ni1-S5: 2.1323(7), Ni1-S6: 2.1115(9); C1-S1: 1.720(3), C2-S2: 1.708(3), C17-S5: 1.720(3), C18-S6: 1.699(3); C1-C2: 1.400(4), C17-C18: 1.400(4); S1-Ni1-S2: 90.61(3), S6-Ni1-S5: 90.80(3); S1-C1-C2-S2: -5.7(3), S5-C17-C18-S6: 1.4(3); (b) Ni1-S1: 2.1131(17), Ni1-S2: 2.1246(16), Ni1-S5: 2.1626(16), Ni1-S6: 2.1647(16), Ni2-S8: 2.1125(16), Ni2-S9: 2.1147(19), Ni2-S12: 2.1716(16), Ni2-S13: 2.1683(19); C1-S1: 1.714(5), C2-S2: 1.706(5), C17-S5: 1.678(5), C18-S6: 1.690(5), C26-S8: 1.712(6), C27-S9: 1.710(6), C42-S12: 1.678(6), C43-S13: 1.699(5); C1-C2: 1.372(9), C17-C18: 1.393(8), C26-C27: 1.385(9), C42-C43: 1.396(8); S1-Ni1-S2: 90.92(6), S6-Ni1-S5: 94.53(6), S8-Ni2-S9: 90.47(7), S12-Ni2-S13: 93.97(6); S1-C1-C2-S2: 1.2(6), S5-C17-C18-S6: 2.6(7), S8-C26-C27-S9: -2.5(6), S12-C42-C43-S13: 5.5(7).

Table S3: Average core bond lengths for Ni(C_4dtpdt)(*i*Pr_2timdt) (**2**), compared to those of Ni(C_4dtpdt)$_2$ (**1a**) and Ni(*i*Pr_2timdt)$_2$ (**1b**).[19] For the errors values on the average bond lengths, both the internal and external errors were assessed, and the stated value represents the larger of the two. Differences in bond length lying within their error margin are shown in italics.

Bond	Bond Lengths / Å			
	**1a**	**1b**	**2**	Δ**2**/**1**(**a**/**b**)
CC(*i*Pr_2timdt)	-	1.385(7)	1.394(6)	+0.010(9)
CC(C_4dtpdt)	1.400(3)	-	1.378(7)	-0.022(7)
CS(*i*Pr_2timdt)	-	1.695(5)	1.686(9)	*-0.009(10)*
CS_alkyl(C_4dtpdt)	1.704(5)	-	1.708(2)	*0.004(5)*
CS_aryl(C_4dtpdt)	1.720(3)	-	1.713(5)	-0.007(5)
NiS(*i*Pr_2timdt)	-	2.158(2)	2.167(4)	+0.008(4)
NiS_alkyl(C_4dtpdt)	2.115(5)	-	2.120(5)	*0.005(6)*
NiS_aryl(C_4dtpdt)	2.129(4)	-	2.1128(12)	-0.016(4)

Film Characterization

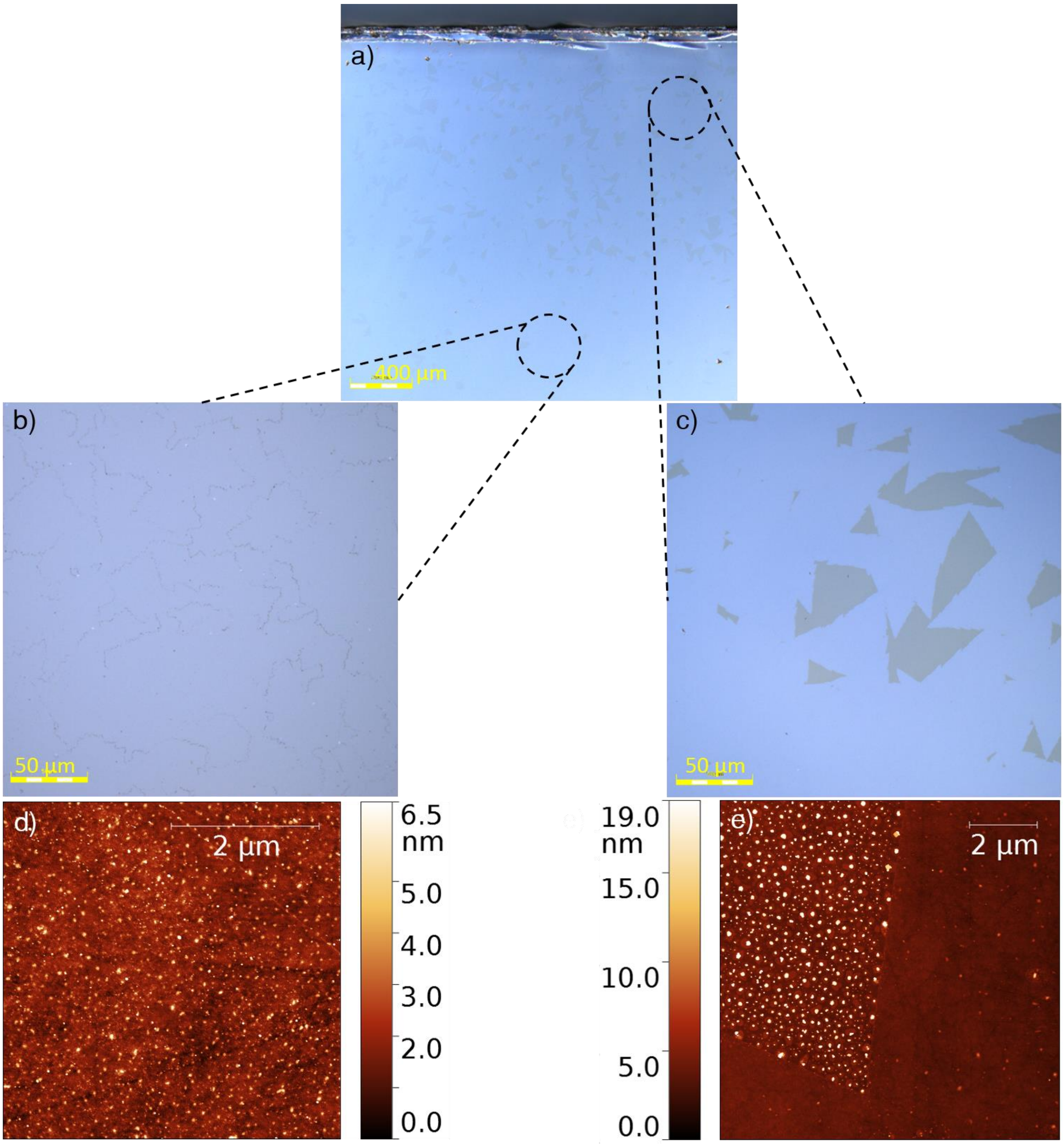

Figure S5: Characterization of as received MoS_2 monolayers on c-cut sapphire wafers. (a – c) Optical microscopy showing gradient of coverage from edge (a), with coalesced grains in the center of the wafer (b) and incomplete coverage at the edge (c); (d – e) AFM images of monolayer surface (d) and step edge of monolayer flake (e). Expanded areas are for illustrative purposes, and do not necessarily correspond to the area measured.

Note: The monolayer coverage of the as received films was generally very high. However, incomplete monolayer coverage was observed on some edges of the wafers that extended for <2 mm from the edge (Figs. S5a & S5c). The surface of the MoS_2 was not very smooth, with a high density of isolated features (Fig. S5d), measuring up to 100 nm in diameter, and yielding a surface roughness of R_q = 0.55 nm. The surface texture of the exposed wafer was extremely rough (R_q = 3.2 nm) (Fig. S5e), and likely comprised precursors from the CVD growth. The latter features were not observed in the transferred films, suggesting that they were either not delaminated, or they were washed away in the polystyrene removal step. It should be noted that neither the features on the wafer, nor on the MoS_2, were noticeably changed upon annealing at 250°C, or soaking in DCM.

While absorption spectroscopy and XPS was performed in the center of the films, thereby avoiding the sub-monolayer regions, the films transferred to devices were not preferentially oriented, and some uncovered regions could be seen over the electrode arrays (*c.f.* Fig. S8c).

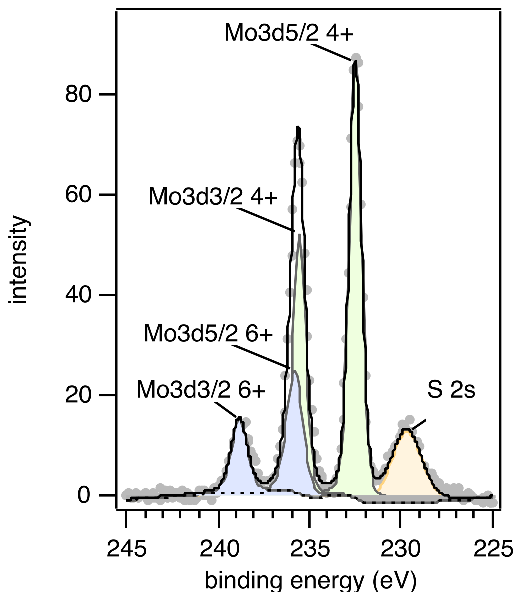

Figure S6: Example of peak fitting from a representative XPS signal measured on MoS_2 displaying the Mo 3d and S 2s core level fitting.

Note: The XPS signal from MoS_2, MoS_2-C, MoS_2-**1b** and MoS_2-**2** was measured and fitted according to Figure S6 in order to quantify the elemental amount ratios in the samples. It should be noted that all MoS_2 films showed non-negligible charging during measurements. Furthermore, upon prolonged measurement, the spectral shape showed a gradual change. This suggests that MoS_2 is sensitive to either the X-ray irradiation, or the local heating under ultra-high vacuum. Therefore, all measurements were recorded over a standardized measurement time of 2 hrs to limit the damage to the film, and to achieve comparable results.

Table S4: S 2s and Mo 3d5/2 peak areas and the S/Mo ratios according to the fit of the XPS displayed in Figure S6.

	**S 2s**	**Mo 3d5/2**	**S/Mo**
MoS_2	30.24	42.25	1.40
MoS_2-**2**	66.28	118.30	1.78
MoS_2-C	25.61	33.94	1.33
MoS_2-**1b**	166.32	224.14	1.35

Device Fabrication and Testing

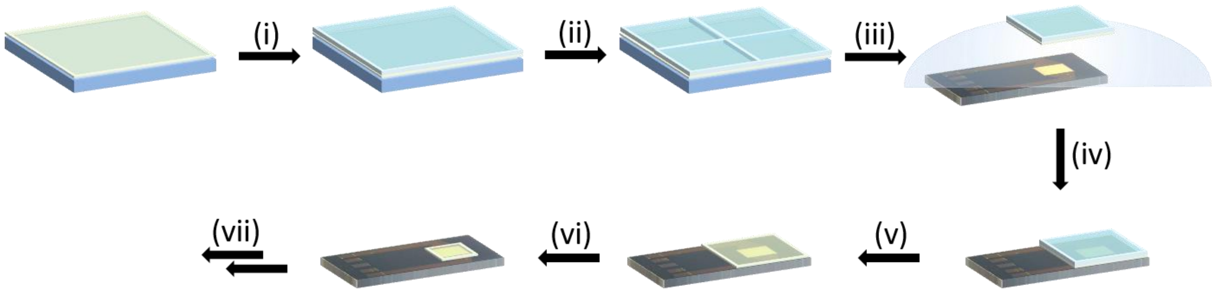

Figure S7: Processing steps of MoS_2 devices by surface-energy-assisted transfer:[13] (i) spin coating of sapphire-supported MoS_2 with polystyrene; (ii) dicing the film into 5x5 mm sections; (iii) release of MoS_2 film with water; (iv) transfer to IDE array of transistor substrate; (v) removal of polystyrene with toluene; (vi) pixel isolation with toluene-soaked cotton bud; (vii) further processing steps, if required.

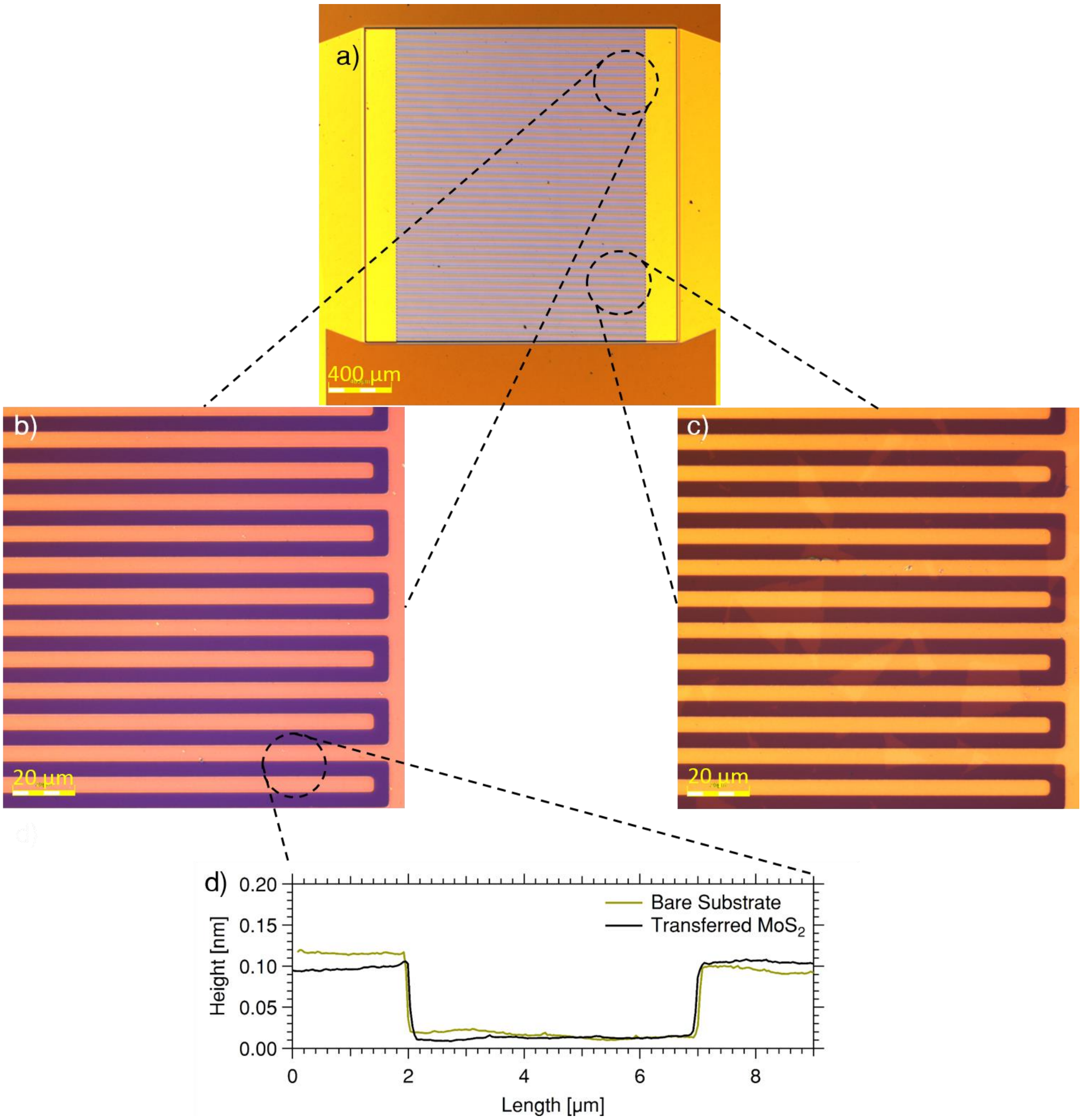

Figure S8: Microscopy of representative processed devices (channel length L = 5 µm, W = 32 cm). (a – b) Optical microscopy of the MoS_2 film after transfer to IDE array (a), showing, in general, high coverage of MoS_2 (b), with some incomplete coverage in regions (paler color on dielectric) for some devices (c); (d) AFM of the electrode gap for a transferred film, compared to the bare electrode array. Expanded areas are for illustrative purposes, and do not necessarily correspond to the area measured.

Note: Even for unannealed devices, the MoS_2 followed the contours of the electrodes, suggesting collapse to happen during the drying process, rather than upon post-processing.

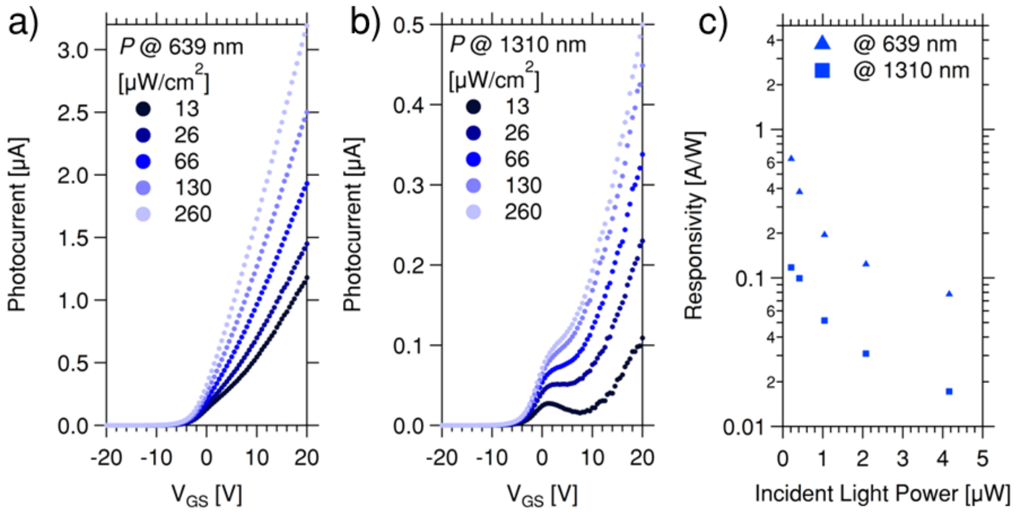

Figure S9: Optoelectronic characterization of a IDE BGBC MoS_2-**2** phototransistor (channel length L = 5 μm): (a – b) photocurrent response (I_{photo} = I_{light} - I_{dark}) as a function of gate voltage and illumination intensity at 639 nm (a) and 1310 nm (b) (V_{DS} = 1 V); (c) responsivity (R = I_{photo}/(P_{opt} x A) where A = device area (W x L) = 1.6 x10^{-2} cm^2) extracted from (a) and (b) at V_{GS} = 0V.

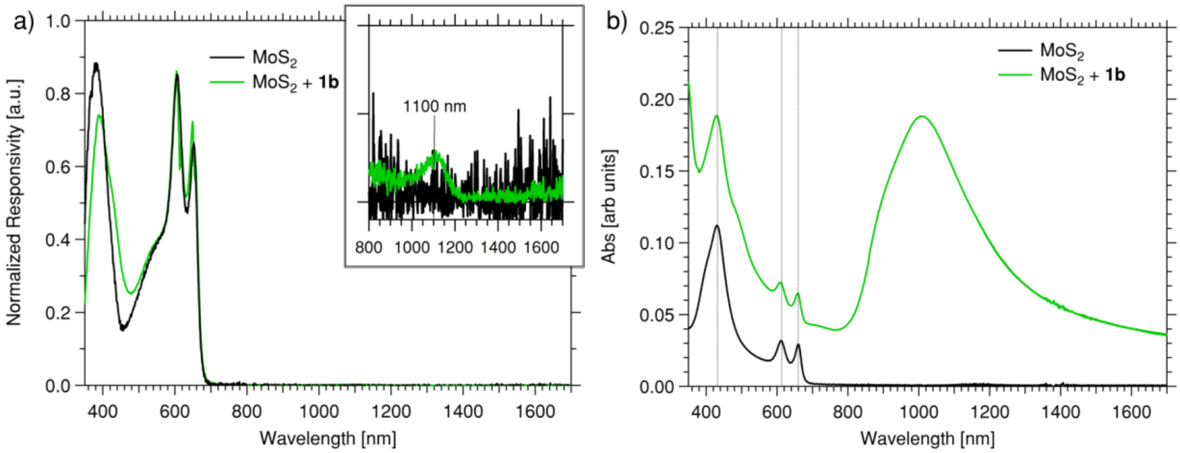

Figure S10: (a) Photocurrent action spectrum of IDE BCBG MoS_2 devices (V_{DS} = 10 V, V_{GS} = 0 V) with and without a drop coated film of **1b** (normalized the B excitonic process); (inset: magnified NIR region re-measured under higher light power and showing a small contribution from **1b** to the photocurrent at *ca*. 1100 nm; (b) absorption spectrum of MoS_2 with similar drop coated film of **1b**, compared to as received MoS_2.

REFERENCES

[1] Bigoli, F.; Deplano, P.; Devillanova, F. A.; Ferraro, J. R.; Lippolis, V.; Lukes, P. J.; Mercuri, M. L.; Pellinghelli, M. A.; Trogu, E. F.; Williams, J. M. Syntheses, X-Ray Crystal Structures, and Spectroscopic Properties of New Nickel Dithiolenes and Related Compounds. *Inorg. Chem.* **1997**, *36*, 1218–1226.

[2] Elderfield, R. C.; Covey, I. S.; Geidushek, J. B.; Meyer, W. L.; Ross, A. B.; Ross, J. H. Synthesis of potential anticancer agents. I. Nitrogen Mustards Derived From p-N,N-bis(2-chloroethyl)-aminobenzaldehyde. *J. Org. Chem.* **1958**, *23*, 1749–1753.

[3] Li, Q.; Rukavishnikov, A. V.; Petukhov, P. A.; Zaikova, T. O.; Keana, J. F. W. Nanoscale 1,3,5,7-Tetrasubstituted Adamantanes and *p*-Substituted Tetraphenyl-methanes for AFM applications. *Org. Lett.* **2002**, *4*, 3631–3634.

[4] Monnereau, C.; Blart, E.; Odobel, F. A Cheap and Efficient Method for Selective *para*-Iodination of Aniline Derivatives. *Tet. Lett.* **2005**, *46*, 5421–5423.

[5] Gareau, Y.; Beauchemin, A. Free-Radical Reaction of Diisopropyl Xanthogen Disulphide with Unsaturated Systems. *Heterocycles* **1998**, *48*, 2003–2017.

[6] Data Collection and Processing Software, Rigaku Corporation (1998–2015). Tokyo 196–8666, Japan.

[7] Altomare, A.; Cascarano, G.; Giacovazzo, C.; Guagliardi, A. Completion and Refinement of Crystal Structures with *SIR*92. *J. Appl. Cryst.* **1993**, *26*, 343–350.

[8] Crystal Structure Analysis Package, Rigaku Corporation (2000-2017). Tokyo 196–8666, Japan.

[9] Sheldrick, G. M. Crystal Structure Refinement with SHELXL. *Acta Cryst*. **2015**, *C71*, 3–8.

[10] Spec, A. L. Structure Validation in Chemical Crystallography. *Acta Cryst*. **2009**, *D65*, 148–155.

[11] Sim, D. M.; Kim, M.; Yim, S.; Choi, M.-J.; Choi, J.; Yoo, S.; Jung, Y. S. Controlled Doping of Vacancy-Containing Few-Layer MoS_2 *via* Highly Stable Thiol-Based Molecular Chemisorption. *ACS Nano* **2015**, *9*, 12115–12123.

[12] Gwyddion Free SPM Data Analysis Software Version 2.52 (www.gwyddion.net).

[13] Gurarslan, A.; Yu, Y.; Su, L.; Yu, Y.; Suarez, F.; Yao, S.; Zhu, Y.; Ozturk, M.; Zhang, Y.; Cao, L. Surface-Energy-Assisted Perfect Transfer of Centimeter-Scale Monolayer and Few-Layer MoS_2 Films onto Arbitrary Substrates. *ACS Nano* **2014**, *8*, 11522–11528.

[14] Lim, B. S.; Fomitchev, D. V.; Holm, R. H. Nickel Dithiolenes Revisited: Structures and Electron Distribution from Density Functional Theory for the Three-Member Electron-Transfer Series $[Ni(S_2C_2Me_2)_2]^{0,1-,2-}$. *Inorg. Chem.* **2001**, *40*, 4257–4262.

[15] Chastrette, M.; Rajzmann, M.; Chanon, M.; Purcell, K. F. Approach to a General Classification of Solvents Using a Multivariate Statistical Treatment of Quantitative Solvent Parameters. *J. Am. Chem. Soc.* **1985**, *107*, 1–11.

[16] Kamlet, M. J.; Abboud, J. L.; Taft, R. W. The Solvatochromic Comparison Method. 6. The π* Scale of Solvent Polarities. J. Am. Chem. Soc. **1977**, *99*, 6027–6038.

[17] Warashira, T.; Hoshino, H. Solvent Effects for Spectroscopic Properties of Near-Infrared Absorbing Nickel–Dithiolene Complex [Ni(*i*Pr_2timdt)$_2$] (*i*Pr_2timdt: Monoanion of 1,3-Diisopropylimidazolidine-2,4,5-trithione). *Bull. Chem. Soc. Jap.* **2016**, *89*, 836–841.

[18] Deplano, P.; Pilia, L.; Espa, D.; Mercuri, M. L.; Serpe, A. Square-Planar d^8 Metal Mixed-Ligand Dithiolene Complexes as Second Order Nonlinear Optical Chromophores: Structure/Property Relationship. *Coord. Chem. Rev.* **2010**, *254*, 1434–1447.

[19] Bigoli, F.; Deplano, P.; Devillanova, F. A.; Lippolis, V.; Lukes, P. J.; Mercuri, M. L.; Pellinghelli, M. A.; Trogu, E. F. New Neutral Nickel Dithiolene Complex Derived from 1,3-Dialkylimidazolidine-2,4,5-trithione, Showing Remarkable Near-IR Absorption. *J. Chem. Soc., Chem. Commun.* **1995**, 371–372.
